# The NO Answer for Autism Spectrum Disorder

**DOI:** 10.1002/advs.202205783

**Published:** 2023-05-22

**Authors:** Manish Kumar Tripathi, Shashank Kumar Ojha, Maryam Kartawy, Wajeha Hamoudi, Ashwani Choudhary, Shani Stern, Adi Aran, Haitham Amal

**Affiliations:** ^1^ Institute for Drug Research School of Pharmacy Faculty of Medicine The Hebrew University of Jerusalem Jerusalem 91120 Israel; ^2^ Sagol Department of Neurobiology Faculty of Natural Sciences University of Haifa Haifa 31905 Israel; ^3^ Neuropediatric Unit Shaare Zedek Medical Center Jerusalem 91031 Israel; ^4^ Faculty of Medicine The Hebrew University of Jerusalem Jerusalem 91120 Israel

**Keywords:** autism spectrum disorder, behavior, contactin‐associated protein‐like2, nitric oxide, Shank3, S‐nitrosylation

## Abstract

Autism spectrum disorders (ASDs) include a wide range of neurodevelopmental disorders. Several reports showed that mutations in different high‐risk ASD genes lead to ASD. However, the underlying molecular mechanisms have not been deciphered. Recently, they reported a dramatic increase in nitric oxide (NO) levels in ASD mouse models. Here, they conducted a multidisciplinary study to investigate the role of NO in ASD. High levels of nitrosative stress biomarkers are found in both the Shank3 and Cntnap2 ASD mouse models. Pharmacological intervention with a neuronal NO synthase (nNOS) inhibitor in both models led to a reversal of the molecular, synaptic, and behavioral ASD‐associated phenotypes. Importantly, treating iPSC‐derived cortical neurons from patients with SHANK3 mutation with the nNOS inhibitor showed similar therapeutic effects. Clinically, they found a significant increase in nitrosative stress biomarkers in the plasma of low‐functioning ASD patients. Bioinformatics of the SNO‐proteome revealed that the complement system is enriched in ASD. This novel work reveals, for the first time, that NO plays a significant role in ASD. Their important findings will open novel directions to examine NO in diverse mutations on the spectrum as well as in other neurodevelopmental disorders. Finally, it suggests a novel strategy for effectively treating ASD.

## Graphical abstract

This novel work reveals for the first time that nitric oxide (NO) plays a significant role in autism spectrum disorder (ASD) development. The findings will open future directions in order to examine NO in diverse mutations on the spectrum as well as in other neurodevelopmental disorders and psychiatric diseases. Finally, it suggests a novel strategy for effectively treating ASD.

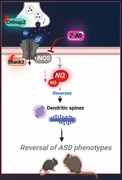

## 1 Introduction

Autism spectrum disorder (ASD) is a serious neurodevelopmental and behavioral disorder;^[^ ^1^ ^]^ it is one of the most disabling conditions and chronic illnesses in childhood. It is characterized by abnormalities in social interactions, deficits in communication, restricted interests, and repetitive behavior.^[^ ^2^ ^]^ Globally, recent epidemiological studies have referred to ASD prevalence rates of 1 in 44 children.^[^ ^3^ ^]^ The number of people with ASD has been growing rapidly. Thus, the prevalence rate has increased nearly threefold over the last 20 years in the US alone. Unfortunately, to date, no effective pharmacological treatments or preventive measures are currently available for effectively managing this spectrum disorder.^[^ ^4^ ^]^ ASD is a highly genetic pathology.^[^ ^5^, ^6^, ^7^, ^8^ ^]^ Rare de novo mutations of genes such as SH3 and multiple ankyrin repeat domains 3 (*SHANK3)*, the contactin‐associated protein‐like2 gene (*CNTNAP2)*, and others appear to have distinct functional effects on the protein‐coding regions of the genome, representing a high risk of developing ASD.^[^ ^9^, ^10^ ^]^

Shank3 is a scaffolding protein located in the postsynaptic density complex of excitatory synapses.^[^ ^11^ ^]^ It binds to neuroligins and actin and participates in regulating actin polymerization, growth cone motility, dendritic spine morphology, and synaptic transmission.^[^ ^12^ ^]^ Deletions or mutations in the *SHANK3* gene have been found in patients with Phelan‐McDermid Syndrome (PMS). Over 50% of these patients are diagnosed with ASD.^[^ ^13^, ^14^ ^]^ This mutation is also found in ASD patients outside the PMS.^[^ ^15^, ^16^ ^]^ Previous studies on Shank3 mutant mice revealed ASD‐like behaviors, such as impaired social interaction, anxiety‐like behavior, and reduced interest in novel objects.^[^ ^17^, ^18^ ^]^ It is considered as one of the most promising mouse models of ASD to date.^[^ ^16^, ^19^, ^20^ ^]^

The *CNTNAP2* gene encodes a neuronal transmembrane protein member of the neurexin superfamily, which is involved in neuron‐glia interactions and clustering of K+ channels in myelinated axons. It plays a key role in synapse formation and stabilization.^[^ ^21^, ^22^ ^]^ Moreover, it is considered one of the crucial genes in autism; it is responsible for delayed speech‐language development and relatively impaired signaling.^[^ ^23^, ^24^ ^]^ *Cntnap2* knockout mice display reduced stability in newly formed dendritic spines.^[^ ^25^, ^26^, ^27^ ^]^ Loss‐of‐function mutations in this gene have been implicated in ASD and cortical dysplasia–focal epilepsy syndrome.^[^ ^28^ ^]^ Studies of *Cntnap2^(‐/‐)^* mice revealed frequent spontaneous seizures and behavioral patterns typical of ASD, such as stereotypic motor movements, behavioral inflexibility, a reduced time of interaction with other mice in a juvenile playtest, and fewer isolation‐induced ultrasonic vocalizations (distress calls) of the mouse pups to their mothers.^[^ ^25^, ^29^ ^]^ However, the etiology of ASD remains elusive.^[^ ^30^, ^31^ ^]^

Recently, we found elevated levels of nitric oxide (NO) in an ASD mouse model;^[^ ^32^, ^33^, ^34^, ^35^ ^]^ we suggested several mechanisms for the involvement of NO, S‐nitrosylation (SNO), and NO‐mediated posttranslational modification (PTM) in ASD pathology.^[^ ^32^, ^33^, ^36^, ^37^, ^38^, ^39^ ^]^

NO is a small gaseous molecule produced in different organs and tissues, including the central^[^ ^40^ ^]^ and peripheral nervous systems.^[^ ^41^ ^]^ At normal physiological levels, NO production and degradation are balanced and involved in normal cell signaling and in regulating numerous physiological functions.^[^ ^42^, ^43^ ^]^ At higher concentrations, however, NO becomes toxic and leads to abnormal cell signaling and cell death.^[^ ^44^ ^]^ NO can regulate cell signaling by SNO, a form of PTM in which a nitroso group is incorporated into a reactive cysteine thiol, forming a nitrosothiol group.^[^ ^45^ ^]^ In the nervous system, NO regulates synaptogenesis, vesicle trafficking, neuronal migration, plasticity, and more. Aberrant NO production can cause nitrosative stress by tyrosine nitration of proteins (3‐Nitrotyrosine (3‐Ntyr)) and by forming S‐nitrosoglutathione (GSNO), which is a radical recombination between NO and GSH thiyl radicals (RS•); consequently, NO can transform other thiols on peptides and proteins.^[^ ^46^ ^]^ At higher concentrations, NO reacts with superoxide radicals, forms peroxynitrite, and induces cell damage. 3‐Ntyr is a product of the reaction of proteins with peroxynitrite. It is an early diagnostic marker of nitrosative stress in neurological disorders.^[^ ^47^, ^48^, ^49^ ^]^ 3‐Ntyr causes disruption of protein native structure and interferes with the phosphorylation ability of tyrosine.^[^ ^50^ ^]^ It disrupts many cellular signaling pathways, such as tyrosine nitration of synaptophysin (Syp), which leads to cholinergic dysfunction.^[^ ^51^, ^52^ ^]^ The free form of 3‐Ntyr is equally toxic.^[^ ^53^ ^]^

**Figure 1:**
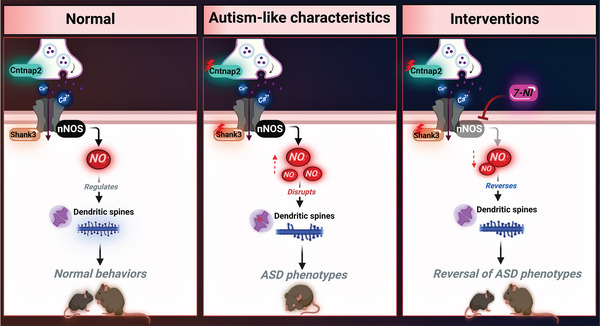
Illustrative figure describing the aberrant role of NO in ASD and the therapeutic potential following the inhibition of nNOS. Under normal conditions, NO acts as a signaling molecule, playing a crucial role in the development and formation of dendritic spines, which are essential for proper cognitive function. However, mutations in the *SHANK3* or *CNTNAP2 (CASPR2)* genes elevate the levels of NO production, which can lead to the disruption of dendritic spines and neuronal functions; this results in behavioral deficits. Pharmacological inhibition of nNOS restores the ASD phenotype.

The dysregulation of NO is responsible for various neurodevelopmental, neuropsychiatric, and neurodegenerative disorders.^[^ ^33^, ^44^ ^]^ However, the role of NO in ASD remains unknown. To prove the causal effect of NO in ASD pathology, we conducted multidisciplinary experiments using cellular and mouse models, iPSC‐derived cortical neurons from patients with *SHANK3* mutations, as well as clinical plasma samples. Importantly, we found that elevated NO levels contribute to ASD‐like defects, whereas inhibiting its production via nNOS led to its reversal (**Figure** **1**). This study suggests a direct link between NO and ASD, which may lead to the discovery of novel therapeutic targets.

## 2 Results

### 2.1 NO Donor Administration to WT Mice Increased Nitrosative Stress and Reduced the Expression of Synaptic, Glutamatergic, and GABAergic Proteins

**Figure 2:**
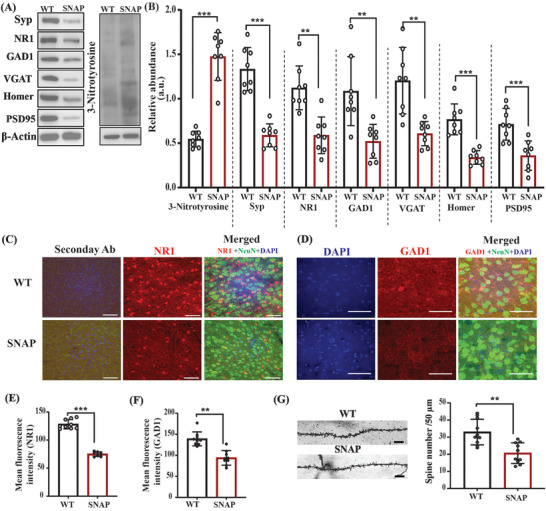
NO donor administration led to nitrosative stress and synaptic pathology in the cortex of WT mice. A) Representative western blots of an indicator of nitrosative stress, 3‐Ntyr, and the synaptic proteins Syp, NR1, GAD1, VGAT, Homer, and PSD95. Β‐actin was used as a reference for protein loading. B) Statistical analysis of the relative abundance of proteins shown in (A). C) Representative confocal images of the NR1, NeuN, DAPI, and their respective secondary ab control fluorescence. The image was captured at 40× magnification and the scale bar = 50 µm. D) Representative confocal images of the GAD1, NeuN, and DAPI fluorescence. The image was captured at 60× magnification and the scale bar = 50 µm. E,F) Statistical analysis of the mean fluorescence intensity of NR1, and GAD1 protein. G) Left—representative images of the dendritic spines; right—statistical analysis of the dendritic spine density. Student's two‐tailed *t*‐test was used for two‐group comparisons. *p < 0.05, **p < 0.01, ***p < 0.001. Groups of mice: WT (*n* = 8); SNAP (WT mice treated with the NO donor compound SNAP, *n* = 8) for western blot analysis and WT (*n* = 9); SNAP (*n* = 9) for the immunofluorescence experiments. In the IF images, red denotes NR1/GAD1, green denotes neuronal marker NeuN, and blue denotes DAPI.

To determine whether NO leads to biochemical and cellular ASD‐like phenotypes, C57BL/6J wild‐type (WT) male mice were treated with an NO donor, *S*‐nitroso‐*N*‐acetyl penicillamine (SNAP, 20 mg kg^−1^) for 10 consecutive days.^[^ ^54^ ^]^ SNAP administration increased NO production, which led to 3‐Ntyr production in the WT+SNAP group, compared with the WT in both the cortex (**Figures** **2**A,B) and the striatum regions (Figure S1A,D, Supporting Information). Next, we determined whether NO affects synaptic protein expression. Importantly, we found that the synaptogenesis biomarker Syp is significantly reduced in SNAP‐treated mice, compared with the WT group in both the cortex (Figure 2A,B) and the striatum (Figure S1A,D, Supporting Information). Furthermore, postsynaptic density protein 95 (PSD95) and Homer levels were decreased in the SNAP‐treated group, compared with the WT group in both the cortex (Figure 2A,B) and the striatum regions (Figure S1A,D, Supporting Information). Next, we tested the glutamatergic and GABAergic biomarkers, which are altered in ASD mouse models. We found a reduction in the NMDA (the NR1 subunit of the *N*‐methyl‐*D*‐aspartate receptor) (NR1) levels following SNAP treatment in the cortex (Figure 2A,B) and the striatum (Figure S1A,D, Supporting Information). Immunofluorescence (IF) analysis of NR1 expression in the somatosensory cortex revealed similar results (Figure 2C,E). GABAergic markers such as glutamate decarboxylase 1 (GAD1) and Vesicular GABA transporter (VGAT) were reduced in the SNAP treatment group, compared with the WT in the cortex (Figure 2A,B) and the striatum (Figure S1A,D, Supporting Information). Furthermore, IF analysis of GAD1 in the somatosensory cortex of mouse brains showed a reduction in the fluorescence intensity in SNAP‐treated mice, compared with the WT (Figure 2D,F). Importantly, we found a significant reduction in the cortical dendritic spine density in SNAP‐treated mice (Figure 2G), similar to that in the ASD mutant mice.

### 2.2 NO Inhibition Led to a Reversal of the Molecular and Synaptic Deficits in the Shank3^Δ4‐22^ (M1) Mouse Model

**Figure 3:**
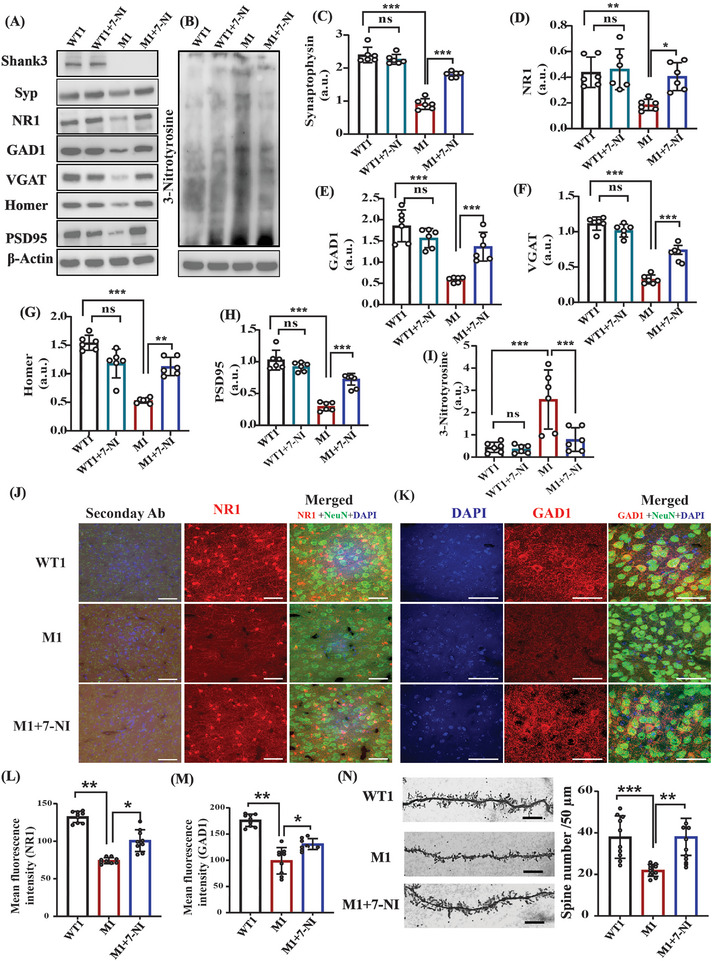
NO inhibition led to a reversal in the molecular and synaptic deficits in the *Shank3^Δ4‐22^* mouse model. A) Representative western blots of synaptic proteins Shank3, Syp, NR1, GAD1, VGAT, Homer, and PSD95 in four groups of mice: WT1, WT1+7‐NI, M1, and M1+7‐NI. *β*‐actin was used as a reference for loading control. B) Representative western blots of an indicator of nitrosative stress; 3‐Ntyr and b‐actin are used as loading control. C) Statistical analysis of the relative abundance of synaptophysin/b‐actin, D) NR1/b‐actin, E) GAD1/b‐actin, F) VGAT/b‐actin, G) Homer/ b‐actin, H) PSD95/b‐actin, I) 3‐Ntyr/b‐actin. J) Representative confocal images of the NR1, NeuN, DAPI, and their relative secondary ab control fluorescence. The image was captured at 40× magnification and the scale bar = 50 µm. K) Representative confocal images of the GAD1, NeuN, and DAPI fluorescence. The image was captured at 60× magnification and the scale bar = 50 µm. L,M) Statistical analysis of the mean fluorescence intensity of NR1 and GAD1 protein shown in (J) and (K), respectively. N) Left—representative images of the dendritic spines; right—statistical analysis of the dendritic spine density. The mean and standard deviation (SD) were calculated for the western blots, immunofluorescence, and spine density counting. A one‐way ANOVA (for IF and spine density) and a two‐way ANOVA test, along with the Bonferroni multiple comparison tests were used for the western blot analyses. *p < 0.05, **p < 0.01, ***p < 0.001, ns = non‐significant. Abbreviations, WT1 (Shank3 WT littermates), WT1+7‐NI (Shank3 WT littermates treated with 7‐NI), M1 (Shank3 mutant mice‐*Shank3^Δ4‐22^*), M1+7‐NI (*Shank3^Δ4‐22^* mice treated with the nNOS inhibitor 7‐NI). The number of mice: WT (*n* = 6), WT1+7‐NI (*n* = 6), M1 (*n* = 6), and M1+7‐NI (*n* = 6). In IF images, red denotes NR1/GAD1, green denotes neuronal marker NeuN, and blue denotes DAPI.

To determine the involvement of NO in ASD, we inhibited its production pharmacologically in the Shank3 mouse (M1) model. For this purpose, M1 mice were treated with neuronal nitric oxide synthase (nNOS) inhibitor, 7‐nitroindazole (7‐NI), for 10 consecutive days. In addition, WT1 mice were treated with 7‐NI as a negative control group (WT1+7‐NI). WT1 and M1 were also treated with their respective vehicles. First, to measure the direct pharmacological effect of 7‐NI in reducing the NO levels, we investigated the key NO byproducts. The results showed that 3‐Ntyr is elevated in M1 mice. Administering 7‐NI (80 mg kg^−1^) to the M1 group led to a reversal of the elevated levels of 3‐Ntyr in both the cortex (**Figure** **3**B,I) and the striatum (Figure S1B,E, Supporting Information). Using the Griess reagent assay, we investigated the NO production in the M1 group and found that nitrite concentrations are increased in the M1 group, whereas treatment with 7‐NI reduces its levels (Figure S2A, Supporting Information). The NO/cGMP pathway is one of the most important NO signaling pathways. Thus, we measured the cGMP levels in the M1 cortex. cGMP levels were increased in M1 mice, but 7‐NI treatment reduced the levels significantly (Figures S2C, Supporting Information); however, no changes were found in the cAMP levels in all groups (Figure S2E, Supporting Information). Next, we measured Syp, which is correlated with synaptogenesis,^[^ ^55^ ^]^ in the M1 group and found a reduction in cortical Syp protein expression, as shown previously.^[^ ^56^, ^57^ ^]^ 7‐NI treatment to M1 mice led to a significantly elevated level of Syp compared with non‐treated M1 mice (Figure 3A,C). Similar results were found in the striatum (Figure S1B,E, Supporting Information). Furthermore, 7‐NI administration significantly increased the levels of Homer and PSD‐95 in the M1 group, compared with WT1 in the cortical (Figure 3A,G,H) and striatal regions (Figure S1B,E, Supporting Information). NR1 protein expression was reduced in the M1 group, compared with WT1, whereas 7‐NI treatment significantly increased its levels in the cortex (Figure 3A,D) and the striatum (Figure S1B,E, Supporting Information). IF analysis of the somatosensory cortex revealed the same results of NR1 protein expression following 7‐NI treatment (Figures 3J,L). Reduced GAD1 and VGAT levels were significantly restored in the M1 group by 7‐NI administration in the cortex (Figure 3A,E,F) and the striatum (Figure S1B,E, Supporting Information). IF of GAD1 in the somatosensory cortex showed similar results (Figure 3K,M). Spine density measurements showed a decrease in the dendritic spine number in the M1 cortical neurons, as found previously,^[^ ^58^ ^]^ compared with the WT1 group, whereas 7‐NI treatment restored the dendritic spine number in the M1 mice (Figures 3N). No changes were found in the expression of all the above‐mentioned proteins in the WT1 group after 7‐NI treatment in the cortex (Figure 3A–I) and the striatum (Figure S3A,B, Supporting Information).

### 2.3 NO Inhibition Led to A Reversal of the Molecular and Synaptic Deficits in the Cntnap2^(‐/‐)^ (M2) Mouse Model

**Figure 4:**
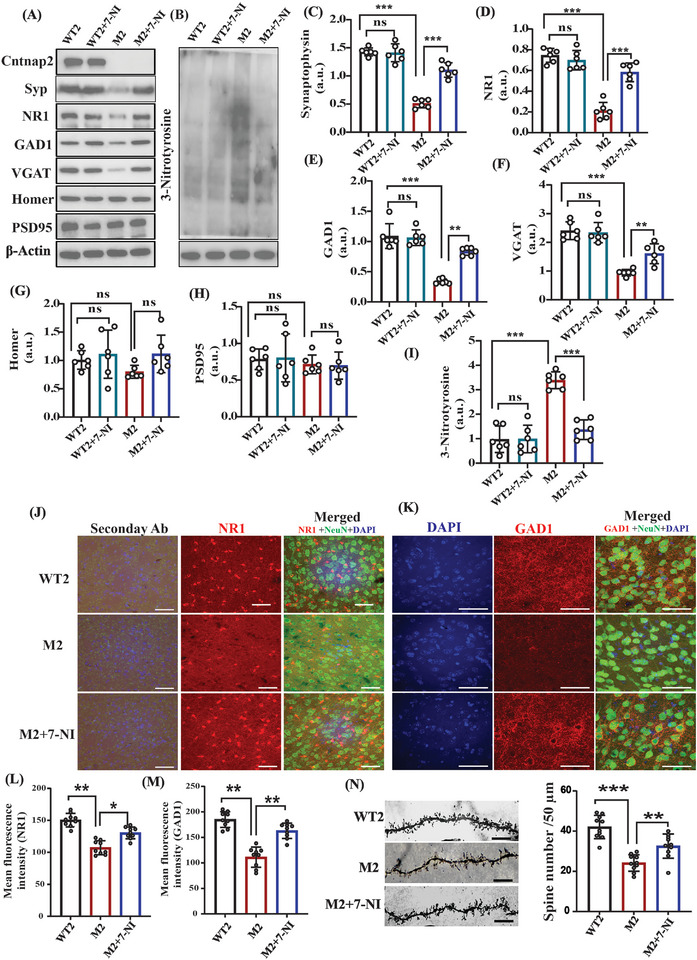
NO inhibition in the Cntnap2^(‐/‐)^ mutant (M2) mouse model led to a reversal of the molecular and synaptic deficits. A) Representative western blots of synaptic proteins Cntnap2, Syp, NR1, GAD1, VGAT, PSD95, and Homer in 4 groups of mice: WT2, WT2+7‐NI, M2, M2+7‐NI (M2 mice treated with 7‐NI). *β*‐actin was used as loading control. B) Representative western blots of an indicator of nitrosative stress, 3‐Ntyr; b‐actin was used as loading control here. C) Statistical analysis of the relative abundance of Synaptophysin/b‐actin. D) NR1/b‐actin, E) GAD1/b‐actin, F) VGAT/b‐actin, G) Homer/b‐actin, H) PSD95/b‐actin, I) 3‐Ntyr /b‐actin. J) Representative confocal images of NR1, NeuN, DAPI, and their relative secondary ab control fluorescence. The image was captured at 40× magnification and the scale bar = 50 µm. K) Representative confocal images of the GAD1, NeuN, and DAPI fluorescence. The image was captured at 60× magnification and the scale bar = 50 µm. L,M) Statistical analysis of the mean fluorescence intensity of NR1, and the GAD1 protein shown in (J) and (K), respectively. N) Left—representative images of the dendritic spines; right—statistical analysis of the dendritic spine density. The mean and standard deviation (SD) were calculated for the western blots, immunofluorescence, and spine density counting. A one‐way ANOVA (for IF and spine density) and a two‐way ANOVA test, along with the Bonferroni multiple comparison tests, were used for the western blot analyses. *p < 0.05, **p < 0.01, ***p < 0.001, ns = non‐significant. Abbreviations, WT2 (C57bL/6 mice were used as a control), WT2+7‐NI (WT2 mice treated with 7‐NI), M2 (Cntnap2^(‐/‐)^ mutant mice), M2+7‐NI (Cntnap2^(‐/‐)^ mutant treated with the nNOS inhibitor 7‐NI). The number of mice: WT2 (*n* = 6), WT2+7‐NI (*n* = 6), M2 (*n* = 6), and M2+7‐NI (*n* = 6). In IF images, red denotes NR1/GAD1, green denotes neuronal marker NeuN, and blue denotes DAPI.

To test a larger spectrum and to strengthen our findings, we used another ASD model, the Cntnap2^(‐/‐)^ mutant mouse (M2) model. Like the M1 model, we treated M2 mice with 7‐NI. The WT2 mice were also treated with 7‐NI as a negative control group (WT2+7‐NI). WT2 and M2 were treated with their respective vehicles. Like the M1 group, we found that the 3‐Ntyr protein levels are elevated in the M2 group, compared with the WT2 group, and that 7‐NI administration reduces its levels in both the cortex (**Figure** **4**B,I) and the striatum (Figure S1C,F, Supporting Information) regions. We also investigated the NO production using the Griess reagent assay and found that the nitrite concentration increased in the M2 group, whereas treatment with 7‐NI reduced its elevated level (Figure S2B, Supporting Information). cGMP levels were found to be increased in M2 mice and 7‐NI treatment reduces its level significantly, but no change was found in cAMP in any group (Figure S2D,F, Supporting Information). M2 mice exhibited reduced Syp expression, whereas 7‐NI treatment of M2 mice increased Syp protein expression in the cortex (Figure 4A,C) and the striatum (Figure S1C,F, Supporting Information). In the M2 group the NR1, GAD1, and VGAT levels were also increased following NO inhibition by 7‐NI in the cortical (Figure 4A,D–F) and the striatal regions (Figure S1C,F, Supporting Information). IF analysis in the somatosensory cortex revealed the same results of NR1 and GAD1 protein expression following 7‐NI treatment (Figures 4J–L). Protein expression of Homer and PSD‐95 was unchanged in both the cortex (Figure 4A,G,H) and the striatum (Figure S1C,F, Supporting Information). In M2 mouse cortical neurons the spine density (the dendritic spine number) levels increased following 7‐NI treatment,^[^ ^59^ ^]^ compared with the M2 group (Figure 4M). No changes were found in the expression of all the above‐mentioned proteins after 7‐NI treatment to the WT1 group (Figures 4A–I).

### 2.4 NO Inhibition Reversed the ASD‐like Behavior in the *Shank3^Δ4‐22^* Mouse Model

**Figure 5:**
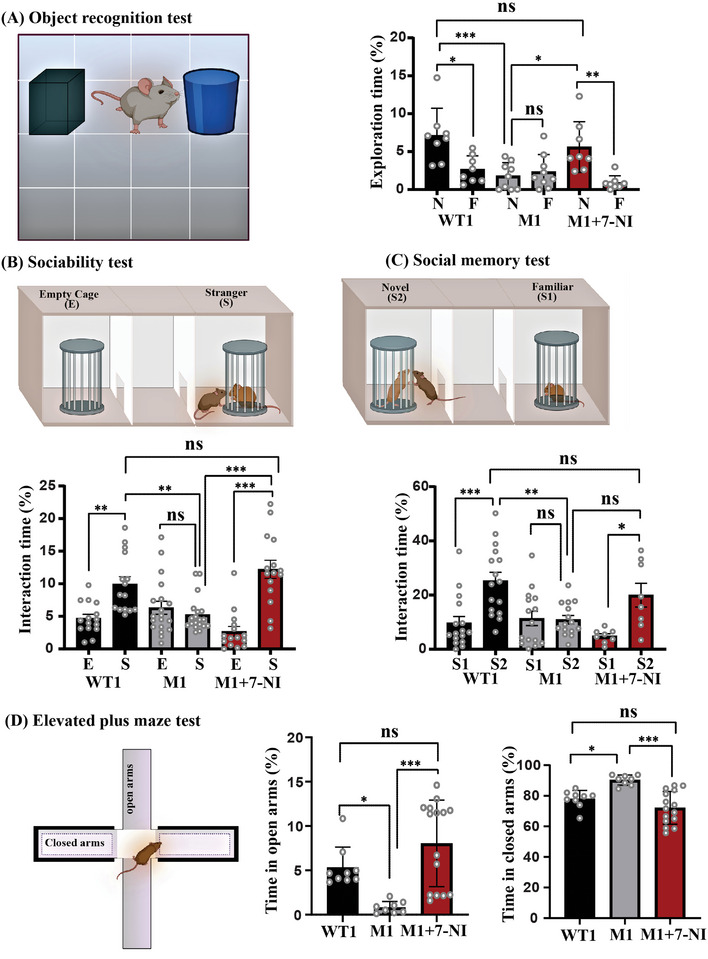
7‐NI administration reversed the ASD‐like behavior in the Shank3 male mice. Behavioral tests analysis was conducted for the following groups of male mice: 1. WT1 (Shank3 littermates treated with vehicle), M1 (Shank3^Δ4‐22^ mice treated with vehicle), and M1+7‐NI (Shank3^Δ4‐22^ treated with 7‐NI (i.p. injection of 80 mg kg^−1^). A) Left—an illustration of Novel object recognition (NOR) test platform. Right—statistical analysis of the object exploration time of either a novel (N) or a familiar (F) object. The WT1 mice spent significantly more time exploring the novel object than the familiar one (*n* = 8, *p = 0.0107). The M1 mice exhibited no significant preference for the novel object over the familiar one, indicating a lack of novelty seeking (*n* = 9, *p* > 0.05). However, the 7‐NI‐treated mutant mice (M1+7‐NI) exhibited a significant preference for the novel object over the familiar object (*n* = 8, **p = 0.0059). B) Upper panel—an illustration of three‐chamber sociability test platform (the first session) and the lower panel—the statistical analysis of the interaction time with either an empty cage (E) or a stranger mouse (S). The WT1 mice spent significantly more time interacting with the stranger mouse than with the empty cage (*n* = 15, **p = 0.0037). The M1 mice did not show any particular interest in engaging in social interaction, indicating reduced sociability among the M1 male mice (*n* = 19, ns = not significant). However, M1+7‐NI mice spent significantly increased time interacting with the stranger mouse than with the empty cage (*n* = 15, ***p < 0.0001). C) Upper panel—an illustration of three‐chamber sociability test platform (the second session) and the lower panel—the statistical analysis of the interaction time with either a familiar mouse (S1) or a novel mouse (S2). The WT1 mice spent significantly more time interacting with S2 than with S1 (*n* = 16, ***p = 0.0004), and the M1 mice showed no particular interest in interacting with S2 or S1(*n* = 15, ns = not significant), whereas the M1+7‐NI mice spent significantly more time interacting with S2 than with S1 (*n* = 8, *p = 0.0432). D) Left—an illustration of the elevated plus maze test platform. Right—statistical analysis of the time spent in the closed arms or in the open arms. The M1 mice spent significantly less time in the open arms compared with the WT1 mice (*p‐value, M1‐WT1 = 0.0484) and WT1+7‐NI (***p‐value M1‐M1+7‐NI = 0.0003). The M1 mice spent significantly more time in the closed arms than the WT1 mice (*p‐value M1‐WT1 = 0.0206). However, the M1+7‐NI mice spent significantly (***p‐value M1‐M1+7‐NI < 0.0001) less time in the closed arms compared with their vehicle‐treated littermates‐M1 (*N* = 9, 8, and 15 for WT1, M1, and M1+7‐NI, respectively). In all tests, the time is presented as the percentage of the total time. The data are presented as the mean ± SD. Statistical significance was determined using a two‐way ANOVA with Bonferroni's multiple comparisons tests. *p < 0.05, **p < 0.001, ***p < 0.0001, and ns = non‐significant.

To correlate our biochemical and cellular findings with the behavioral phenotype, we tested the potential effects of inhibiting NO on the ASD‐like behavior in M1 mice. Both male and female mice were tested separately. Initially, we performed an open field test to examine the motor activity in mice (the distance traveled and the velocity). In the open field test, no significant differences were observed in the distance traveled and the velocity among the groups in both male and female mice (Figure S4A,B, and S7A, Supporting Information). The novel object recognition (NOR) test showed that the WT1 male mice spent significantly more time exploring the novel object than the familiar object, whereas the M1 male mice did not exhibit any significant preference for the novel object over the familiar one. This suggests that the M1 group has deficits in novelty seeking as well as in memory. However, following treatment with 7‐NI, the M1+7‐NI male mice exhibited a significant preference for the novel object over the familiar object (**Figure** **5**A). All three groups (WT1, M1, and M1+7‐NI) of female mice showed significantly more interest in exploring the novel object than the familiar object (Figures S7B, Supporting Information). A three‐chamber sociability test was conducted to evaluate the interest of the mice in social interaction with a stranger mouse. Generally, the mice would be more interested in the stranger mice rather than in an object/empty cage (sociability). In addition, the mice tend to investigate a novel intruder more than a familiar one (social memory). The three‐chamber sociability test is conducted in two different sessions. In the first session, the mice encounter a stranger mouse (S) and an empty cage (E). This session determines the sociability of the mice (Figure 5B). In the second session, the mice encounter the first intruder (S1) and a novel intruder (S2). This second session determines the social memory (Figure 5C).

The analysis revealed that the WT1 male mice spent significantly more time interacting with the stranger mouse than with the empty cage, whereas the M1 mice did not show any particular interest in either of the two cases, indicating reduced sociability in the M1 male mice. However, the M1+7‐NI mice spent significantly increased time interacting with the stranger mouse than with the empty cage (Figures 5B). Interestingly, none of the female groups showed a particular interest in interacting with either the stranger mouse or the empty cage (Figures S7C, Supporting Information). In the social memory test, the M1 mice showed no particular interest in interacting with the novel mouse than with the familiar one, whereas the M1+7‐NI mice, like the WT1 male mice, spent significantly more time interacting with the novel mouse than with the familiar mouse (Figures 5C). The female WT1 mice spent a significantly prolonged interaction time with the novel mouse than with the familiar one. Interestingly, both the vehicle‐treated M1 female mice and the M1+7‐NI female mice did not show any significant difference in the interaction time in both the novel and familiar mice (Figures S7D, Supporting Information).

Next, the elevated plus maze test was performed to evaluate the anxiety‐like behaviors in the mice. Typically, anxiety is defined as the time spent in either closed or in open arms. The analysis revealed that the M1 male mice spent significantly less time in the open arms compared with their WT1 male littermates, whereas following the inhibition of NO, the M1+7‐NI male mice spent a significantly prolonged time in the open arms compared with their vehicle‐treated M1 male counterparts (Figures 5D). Analysis of the time spent in closed arms showed increased anxiety behaviors among the M1 mice, and following treatment with 7‐NI, the MI mice spent significantly less time in the closed arms than did the vehicle‐treated M1 mice (Figures 5D). The female mice in all tested groups showed no significant difference in the time spent in either the closed or opened arms (Figures S7E, Supporting Information). The discrimination index for the different behavioral tests was calculated for all groups of both male and female mice (Figures S10 and S11, Supporting Information). No change was found in the behavior of WT1 male mice following 7‐NI treatment (Figures S5, Supporting Information). Collectively, these results suggest that the autistic behavioral phenotypes observed in Shank3^Δ4‐22^ mice can be reversed by inhibiting neuronal NO production.

### 2.5 NO Inhibition Reversed the ASD‐Like Behavior in the Cntnap2^(‐/‐)^ (M2) Mouse Model

**Figure 6:**
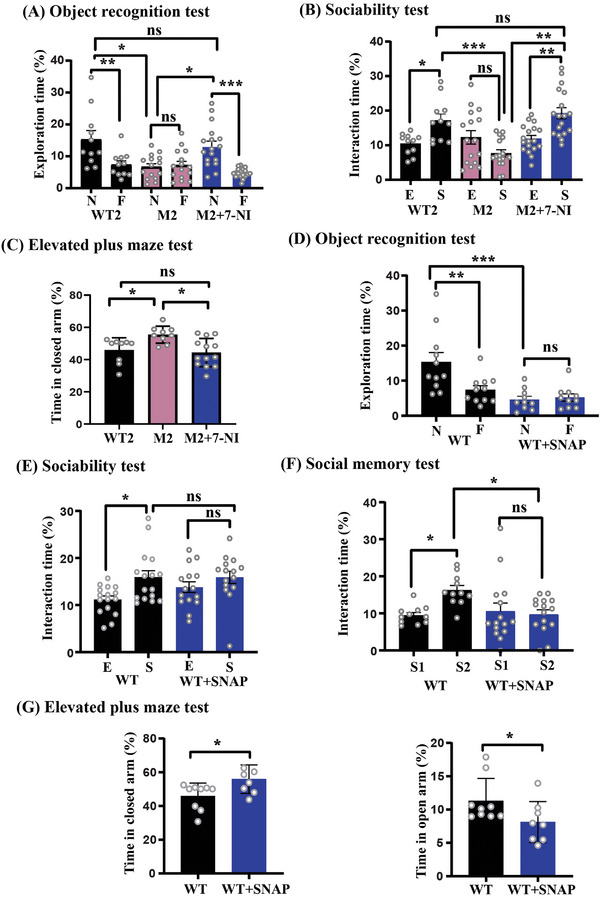
NO inhibition reversed the autistic behavior abnormalities in Cntnap2^(‐/‐)^ mutant male mice, whereas NO donor treatment led to behavioral abnormalities in WT male mice. A–C) Behavioral test analyses for the following groups of male mice: 1) WT2 (wild‐type mice treated with vehicle), 2) M2 (Cntnap2^(‐/‐)^ mice treated with vehicle), and 3) M2+7‐NI (Cntnap2^(‐/‐)^ treated with 7‐NI) (i.p. injection of 80 mg kg^−1^). D–G) Analysis of the WT (C57BL/6J or black6) male mice and WT mice treated with SNAP (20 mg/kg). A) Novel Object recognition test (NOR). The WT2 mice spent significantly more time exploring the novel (N) object than the familiar (F) one (*n* = 11, **p = 0.0090). The M2 mice did not show any particular interest in exploring either the novel object or the familiar one, indicating a lack of novelty seeking and interest (*n* = 15, ns = not significant). However, M2+7‐NI exhibited significantly increased time exploring the novel object than the familiar one (*n* = 17, ***p = 0.0002). B) Three‐chamber sociability test. The WT2 mice spent a significantly prolonged time interacting with the stranger mouse than with the empty cage (*n* = 11, *p = 0.0202). The M2 mice did not show any particular interest in engaging in social interaction, indicating reduced sociability among the M2 male mice (*n* = 16, *p* = 0.3313). However, the M2+7‐NI mice spent significantly increased time interacting with the stranger mouse than with the empty cage (*n* = 18, **p = 0.0024). C) Elevated plus maze test. The M2 mice (*n* = 9) spent significantly more time in the closed arms than did the WT2 mice (*n* = 9, *p = 0.0371), showing increased anxiety among the M2 mice. However, the M2+7‐NI mice spent significantly (*n* = 13, **p = 0.0067) less time in the closed arms than did the M2 mice. In all tests, the time is presented as the percentage of the total time. The data are presented as the mean ± SD. Statistical significance was determined using a two‐way ANOVA with Bonferroni's multiple comparisons tests. *p < 0.05, **p < 0.001, ***p < 0.0001, and ns = non‐significant. D) Novel object recognition test showing the normal spontaneous behaviors of the WT mice in exploring the novel object rather than the familiar one (*n* = 11, *p = 0.0139). The WT+SNAP mice failed to display a significant preference either for the novel object or for the familiar one (*n* = 10, *p* = 0.6910). E) The time interacting with either E or S. The WT mice spent significantly more time with S than with E (*n* = 16, *p = 0.0051). In contrast, the WT+SNAP mice did not show any particular interest in interacting with S or E (*n* = 15, *p* = 0.2387). F) The WT mice spent more time interacting with the S2 than with S1 (*n* = 11, ***p  =  0.0001), whereas the WT+SNAP mice did not show any significant preference to engage in social interaction with S1 or S2 (*n* = 15, *p* = 0.7452). G) Left—the time spent in closed arms. The WT+SNAP mice (*n* = 8) spent significantly more time in the closed arms compared with their WT counterparts (*n* = 9, *p = 0.0221). G) Right—the time spent in open arms shows that the WT+SNAP mice spent significantly less time in the open arms than did the WT mice (*p = 0.0310). In all tests, the time is presented as the percentage of the total time. The data are presented as the mean ± SD. A Two‐tailed *t*‐test was conducted to determine the statistical significance. *p < 0.05, **p < 0.001, ***p < 0.0001, and ns = non‐significant.

Following our behavioral findings of the Shank3 mouse model, we tested the same behavioral parameters in the Cntnap2^(‐/‐)^ (M2) mouse model. In all the experiments the male and female mice were tested. The open field test revealed a non‐significant difference in the distance traveled and the velocity among the tested groups in both sexes (Figures S4C,D,S8A,B, Supporting Information). The NOR test showed that the WT2 male mice spent significantly more time exploring the novel object than the familiar one, whereas the M2 male mice showed no significant preference for either the novel object or the familiar one (**Figures** **6**A). However, following treatment with 7‐NI, the M2+7‐NI male mice spent significantly more time exploring the novel object than the familiar one (Figures 6A). Similar results were found in the female mice (Figures S8C, Supporting Information).

Next, we performed a three‐chamber sociability test that revealed a significantly increased interaction time with the stranger mouse than with the empty cage in WT2 mice. M2 male mice did not show any significant preference to interact with either the empty cage or the stranger mouse, reflecting sociability deficits among the M2 mice. However, the M2+7‐NI male mice spent significantly more time interacting with the stranger mouse than with the empty cage (Figures 6B). None of the female groups showed a significant preference for interacting with either the stranger mouse or the novel mouse in two different tests (Figure S8D,E, Supporting Information, respectively).

The elevated plus maze test showed that the M2 male mice spent significantly more time in the closed arms compared with their WT2 littermates and compared with the M2+7‐NI group (Figures 6C). The time in the open arms analysis showed that the M2 female mice spent significantly less time in the open arms compared with the WT2 female mice. However, the M2+7‐NI female mice spent significantly increased time in the open arms compared with the vehicle‐treated M2 female mice (Figures S8F, Supporting Information). The M2 female mice spent significantly increased time in the closed arms compared with the WT2 female mice. Moreover, following treatment with 7‐NI, the M2+7‐NI female mice did not show any significant change in their time spent in the closed arms (Figures S8G, Supporting Information). Furthermore, in the negative control experiments, no significant changes were observed in mouse behavior between the WT2+7‐NI and the WT2 mice (Figures S5, Supporting Information). All together, these results prove that inhibiting the NO production attenuates and reverses the ASD phenotypes in Cntnap2^(‐/‐)^ male mice.

### 2.6 NO Donor Administration Induced ASD‐Like Behavior in WT Mice and Enhanced the ASD Phenotype in Mutant Mice

To examine the potential effects of elevated NO levels on mouse behavior, a large set of behavioral tests were conducted on the WT and mutant mice. Mice were treated with SNAP as described earlier. Both males and females were tested.

The open‐field test analysis revealed no significant difference in the distance traveled and the velocity between the WT and the WT+SNAP‐treated male mice (Figures S4E,F, Supporting Information). The WT+SNAP female mice showed an increased distance traveled and velocity compared with the WT female mice (Figure S9A,B, Supporting Information). The NOR test showed that the WT male mice spent significantly more time exploring the novel object than the familiar object. In contrast, the WT+SNAP male mice failed to display a preference for the novel object over the familiar one (Figure 6D). Similar results were found in female mice (Figure S9C, Supporting Information). These results indicate a lack of interest in exploring novel objects as well as a disrupted memory in response to higher NO levels. The three‐chamber sociability test showed that the WT mice spent significantly more time interacting with the stranger mouse than with the empty cage, whereas the WT+SNAP male mice did not exhibit any significant preference to interact with either the empty cage or the stranger mouse (Figures 6E). The female mice in both test groups did not show any significant preference for interacting with the stranger mouse or the empty cage (Figure S9D, Supporting Information). The second session of the test (social memory) showed that the WT mice spent significantly more time interacting with the novel mouse (S2) than with the familiar mouse (S1). However, WT+SNAP male mice failed to differentiate between S1 and S2, indicating reduced social memory in response to higher levels of NO (Figure 6F). Similar results were found in female mice (Figure S9E, Supporting Information).

Next, the elevated plus‐maze test was conducted; the analysis revealed that the WT SNAP‐treated male mice spent more time in closed arms compared with the WT mice (Figures 6G—Left). WT+SNAP male mice spent significantly less time in the open arms than did the WT male mice (Figures 6G—Right), reflecting an anxiety‐like behavior among the mice that were treated with SNAP. The WT+SNAP female mice showed results similar to the males in open arms and no significance differences were found in the closed arms in both female groups (Figures S9F,G, Supporting Information). Collectively, these data suggest that high levels of NO may potentially induce ASD‐like behaviors in WT mice.

As a positive control, we examined whether higher levels of NO can aggravate the ASD‐like behavior in M1 and M2 mice. The mutant male mice were also treated with SNAP as described previously and were subjected to the same series of behavioral tests. The NOR test showed no significant difference in time when both M1 and M1+SNAP male mice explored either a novel object or a familiar one (Figures S6A, Supporting Information). Similar results were found in the M2 and M2+SNAP‐treated male mouse group (Figures S6D, Supporting Information). In the three‐chamber sociability test, the M1 and M1+SNAP male mice did not exhibit any significant preference to interact with either the stranger mouse or the empty cage. However, the M1 mice treated with SNAP interacted more with the empty cage than did the vehicle‐treated M1 mice (Figure S6B, Supporting Information). The vehicle‐treated M2 and M2+SNAP male mice spent significantly less time interacting with the stranger mouse than with the empty cage in the three‐chamber sociability test (Figure S6E, Supporting Information). In the elevated plus maze test, no effect of SNAP was observed regarding the M1 and M2 mouse behaviors (Figure S6C,F, Supporting Information).

### 2.7 NO Inhibition Reversed Synaptophysin Expression and Reduced Nitrosative Stress in Primary Cortical Neurons Derived from the Mutant Mouse Model

**Figure 7:**
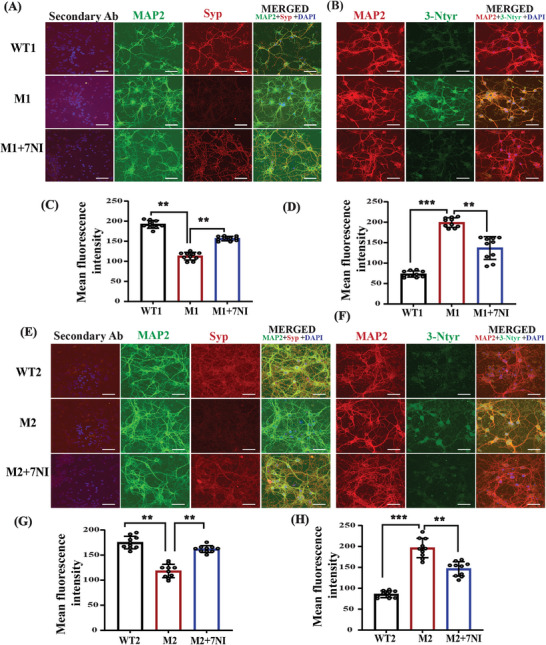
NO contributes to nitrosative stress and reduced synaptogenesis in the primary cortical neurons of the Shank3^Δ4‐22^ and Cntnap2^(‐/‐)^ mutant groups. A) Representative confocal images of the MAP2 (green), Syp (red), DAPI (blue), and their respective secondary ab control in the WT1, M1, and M1+7‐NI groups. B) Representative confocal images of the 3‐Ntyr (green), MAP2 (red), and DAPI (blue) fluorescence in the WT1, M1, and M1+7‐NI groups. C) Statistical analysis of the mean fluorescence intensity of Syp protein. D) Statistical analysis of the mean fluorescence intensity of 3‐Ntyr. E) Representative confocal images of the MAP2 (green), Syp (red), and DAPI (blue) in the WT, M2, and M2+7‐NI mice. F) Representative confocal images of the 3‐Ntyr (green), MAP2 (red), and DAPI (blue) fluorescence in the WT2, M2, and M2+7‐NI groups. G) Statistical analysis of the mean fluorescence intensity of Syp protein. H) Statistical analysis of the mean fluorescence intensity of 3‐Ntyr. All IF images were captured at 60×. The scale bar = 50 µm in scale. A one‐way ANOVA test with the Tukey post hoc test was used for multiple comparisons in all groups. *p < 0.05, **p < 0.01, ***p < 0.001. Abbreviations, WT1 (primary neurons isolated from Shank3 WT embryos), M1 (primary neurons isolated from Shank3^Δ4‐22^ embryos), M1+7‐NI (primary neurons isolated from Shank3^Δ4‐22^ embryos treated with the nNOS inhibitor 7‐NI). WT2 (primary neurons isolated from black6 embryo), M2 (primary neurons isolated from Cntnap2^(‐/‐)^ embryos), M2+7‐NI (primary neurons isolated from Cntnap2^(‐/‐)^ embryos treated with the nNOS inhibitor 7‐NI). WT1 (*n* = 6), M1 (*n* = 6), and M1+7‐NI (*n* = 6), WT2 (*n* = 6) M2 (*n* = 6), and M2+7‐NI (*n* = 6).

To validate our in vivo experiments at the cellular level, we conducted a set of experiments using primary cortical neuronal cell cultures. More specifically, we selectively cultured cortical neurons from the embryos of WT and both mutant groups and treated them with the vehicle and 7‐NI (20 µm), respectively (see the detailed MTT assay in Table S1, Supporting Information). Primary neurons from both mutant groups showed a significant reduction in Syp protein levels, whereas treatment with 7‐NI led to elevated Syp levels (**Figure** **7**A,C,E,G). Furthermore, we found elevated 3‐Ntyr levels in both mutant groups, whereas treatment with 7‐NI reduced the expression of 3‐Ntyr in both mutant groups (Figure 7B,D,F,H), which is consistent with the in vivo findings.

### 2.8 Validating the Key Role of nNOS in *SHANK3* Mutant Cells in

**Figure 8:**
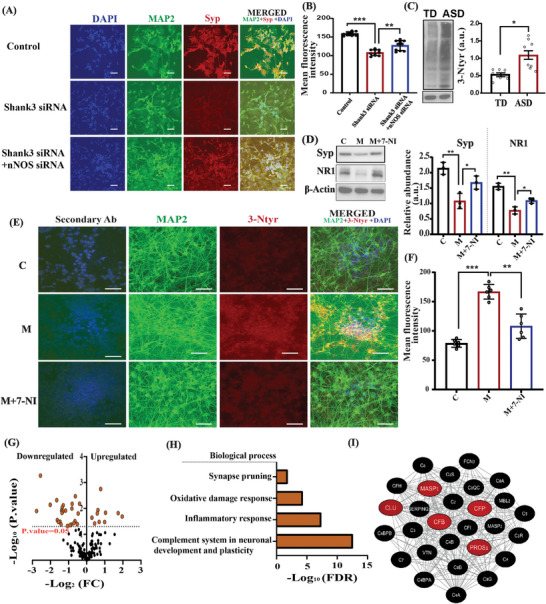
Molecular reprogramming in iPSC‐derived cortical neurons from patients with SHANK3 mutation as well as in human cell lines and blood samples from ASD children. A) Representative confocal images of MAP2 (green), Syp (red), and DAPI (blue) in SHSY5Y (*n* = 10); SHSY5Y+*siSHANK3* (*n* = 10); and SHSY5Y+*siSHANK3+si‐nNOS* (*n* = 10). Image captured at 60×; the scale bar = 50 µm. B) Statistical analysis of the mean fluorescence intensity of Syp protein. Stealth RNAi Negative Control Duplexes was used for the RNA interference (RNAi) experiments as a control for sequence‐independent effects. C) Representative western blot of 3‐Ntyr in human plasma samples in TD (*n* = 8), and ASD (*n* = 8) with statistical analysis. D) Left—a western blot of Syp and NR1 from iPSC‐derived cortical control (C), iPSC‐derived cortical neurons from patients with SHANK3 mutations (Shank3^C.3679insG^) (M), and M+7‐NI (Shank3^C.3679insG^+7‐NI) right—statistical analysis of NR1/actin and Syp/actin. E) Representative confocal image of Ntyr (red) and MAP2 (green) in human iPSC‐derived cortical neurons from control, mutant, and mutant+7‐NI groups. Images were captured at 60× and the scale bar = 50 µm. F) Statistical analysis of the mean fluorescence intensity of 3‐Ntyr shown in (E). G) Volcano plot displaying log 2 (FC) on the *x*‐axis plotted against the −log 10 (*p*‐value) on the *y*‐axis for all the identified proteins that were differentially expressed in the plasma of TD (*n* = 11) and ASD (*n* = 11) individuals. The horizontal line represents a significance level of *p*‐value =  0.05. SNO proteins that were significantly upregulated in ASD (*p* < 0.05) are denoted in orange on the right upper side of the plot, whereas the proteins that were significantly downregulated are denoted in orange on the left upper side of the plot (*p* < 0.05). H) BP and pathways analysis were conducted on the identified significant SNO proteins using the STRING database, version 11.5. Each bar represents the −log 10 (FDR). I) Clustering analysis of the plasma SNO‐proteins. “The complement system in neuronal development and plasticity” was enriched (the number of proteins = 28, FDR = 6.26e‐28). The red nodes are the proteins that were significantly changed between the ASD and TD individuals (*p* < 0.05). STRING, version 11.5, and Cytoscape software version 3.3.0 was used to generate this figure. The data are presented as the mean ± SD. A Two‐tailed *t*‐test was conducted to determine the statistical significance of the comparison between two groups. A one‐way ANOVA test with the Tukey post hoc test was used for multiple comparisons in all groups *p < 0.05, **p < 0.001, ***p < 0.0001, and ns = non‐significant.

To translate our animal research into human data, we conducted a set of experiments using human SHSY5Y cells, iPSC‐derived cortical neurons from patients with SHANK3 mutations, and human plasma samples. In order to validate that nNOS plays a key role in the pathology, we knocked down the expression of *SHANK3* and *nNOS* in differentiated neuroblastoma cells (Figure S12A,B, Supporting Information). First, we tested 3‐Ntyr expression and found that the cells with *SHANK3* knockdown led to increased 3‐Ntyr expression, whereas double knockdown of *SHANK3* and nNOS reduced the expression of 3‐Ntyr levels (Figure S13A,B, Supporting Information). Next, we evaluated the expression of Syp and found that *SHANK3* knockdown reduced its expression, whereas double knockdown with *nNOS* and *SHANK3* restored its expression (**Figure** **8**A,B).

### 2.9 nNOS Inhibition Restores the Expression of Key Synaptic Proteins Using iPSC‐Derived Cortical Neurons from Patients with SHANK3 Mutations

Furthermore, we validated our findings in iPSC‐derived cortical neurons from patients with *SHANK3* mutations and tested their 3‐Ntyr and protein expression levels of NR1 and Syp. We found that the Syp and NR1 levels decreased in iPSC‐derived cortical neurons from *SHANK3* patients, whereas treatment with 7‐NI increased their levels significantly (Figures 8D). The 3‐Ntyr level was increased in *SHANK3* iPSC‐derived cortical neurons and treatment with 7‐NI significantly reduced its level (Figures 8E,F).

### 2.10 Elevation of Nitrosative Stress Biomarker and Reprogramming of the SNO‐Proteome in the Blood Samples of ASD Children

Finally, we measured the levels of 3‐Ntyr by western blotting and analyzed the SNO‐proteome in human blood samples. We tested 3‐Ntyr expression in blood plasma taken from 20 typically developed (TD) individuals and 19 autistic individuals (ASD) (ages: 2–8 years) (see Table S2, Supporting Information). We randomly picked eight ASD and eight TD blood samples to conduct the WB analysis. Importantly, we found an increased expression of 3‐Ntyr in ASD, compared with the TD children (Figure 8C and Table S3, Supporting Information).

To investigate whether a reprogramming in the SNO‐proteome occurs in ASD, we conducted SNOTRAP‐based mass spectrometry technology, which enables a global identification and quantification of SNO proteins.^[^ ^32^, ^60^ ^]^ This was followed by large‐scale systems analysis together with bioinformatics to dissect the biological processes and pathways that are possibly affected by SNO. The analysis was performed using human blood samples taken from two groups: 1. Typically developed children (*n* = 11). 2. Low‐functioning autistic children (*n* = 11). We mapped the SNO‐proteome and identified 149 proteins that were differentially SNOed in the blood of low‐functioning autistic children. A volcano plot analysis was conducted to visualize the significant changes that occur among the SNO proteins (Figures 8G). The fold change (log_2_(FC)) was plotted against the level of statistical significance (−log_10_(*p* value)) on the *x*‐ and *y*‐axes, respectively. Statistical significance thresholds of *p* < 0.05 indicate significantly downregulated and upregulated proteins (Figure 8G).

Gene ontology enrichment analysis was conducted to obtain a system‐level insight into their functionalities and to characterize the biological process and signaling pathways that might be affected due to the altered pattern of SNO in the blood plasma of ASD children. Biological process (BP) and pathways analysis (see Table S4, Supporting Information) revealed the enrichment of key processes and pathways related to ASD, which include synapse pruning , inflammatory response , complement systems in neuronal development and plasticity , as well as oxidative damage response (Figures 8H).

We applied functional clustering analysis to get further functional insights into the data. We used the gene ontology (GO) classification system to classify the proteins into different clusters based on their enriched biological processes. SNO‐proteins formed a distinct functional cluster related to the complement system in neuronal development and plasticity , which is correlated with the BP analysis. Further analysis should be done to validate the link between NO and complement systems in ASD pathology (Figure 8I).

## 3 Discussion

Our study is designed to examine the effect of high levels of NO on the development of ASD. This work shows that NO plays a key role in ASD. Importantly, this was confirmed in cellular, animal models, human iPSC‐derived cortical neurons, as well as in clinical samples. Since the molecular mechanisms underlying ASD pathogenesis remain largely unknown, we provided a new mechanism that shows that NO plays a key role in ASD pathology at the molecular, cellular, and behavioral levels. An increase of Ca^2+^ influx in ASD pathology, including in human and mouse models of *Shank3* and Cntnap2^(‐/‐)^, has already been reported.^[^ ^25^, ^61^, ^62^ ^]^ Ca^2+^ activates nNOS, which then leads to massive production of NO.^[^ ^63^ ^]^ Aberrant NO production induces oxidative and nitrosative stress, leading to increased 3‐Ntyr production and aberrant protein SNO. Our data showed an increase in NO metabolites and 3‐Ntyr production in both mouse models of ASD (Shank3^Δ4‐22^, Cntnap2^(‐/‐)^). Increased 3‐Ntyr was found in iPSC‐derived cortical neurons from patients with SHANK3 mutations, *SHANK3* knocked down in SHSY5Y cells, and in human ASD plasma samples. The elevated levels of 3‐Ntyr in our study are consistent with previous postmortem examinations of ASD patients showing the accumulation of this molecule in the brain.^[^ ^64^ ^]^

Abnormal synaptogenesis is a key factor in ASD pathology. One of the biomarkers of synaptogenesis is Syp,^[^ ^65^, ^66^ ^]^ which is prone to nitrosative stress, and NO regulates its expression.^[^ ^67^ ^]^ Previous works showed that once Syp is nitrated, this leads to proteasomal degradation.^[^ ^68^, ^69^ ^]^ In our study we found that administering NO donor reduces the expression of Syp as well as the high levels of NO in Shank3^(Δ4‐22)^ and Cntnap2^(‐/‐)^ mutant mice, leading to similar results. Administering 7‐NI in both mutant models restored the expression of Syp, which directly confirms that NO plays a key role in synaptogenesis in ASD. Similar results were found in the iPSC‐derived cortical neurons from patients with *SHANK3* mutations and in the *SHANK3* and nNOS double knocked‐down SHSY5Y model. Dendritic spines are tiny membranous outgrowths that are the primary sites for excitatory synaptic transmission.^[^ ^70^ ^]^ Spine density measurement also showed a reduction of dendritic spines in NO donor‐treated mice, compared with the WT group, whereas 7‐NI treatment in mutant mice restored its number. In the CNS, dendritic spines affect synaptic connectivity, which is essential for neuronal circuits^[^ ^102^, ^103^ ^]^ and learning.^[^ ^71^ ^]^ Dysregulation of dendritic spine formation has been reported in ASD patients.^[^ ^72^, ^73^ ^]^ Synaptic pruning is a natural process in the brain where extra or unnecessary synapses between neurons are eliminated, which allows the brain to operate more efficiently.^[^ ^74^ ^]^ This process is crucial for proper brain development and function, since it allows the brain to selectively strengthen important neural connections and diminishes those that are not needed.^[^ ^75^ ^]^ Recent studies showed that inhibiting NO production led to neuronal regrowth and that higher NO promotes synaptic pruning, which is similar to our findings.^[^ ^76^ ^]^ Therefore, the restoration of dendritic spine density following 7‐NI treatment indicates that NO is a critical factor in the development of the synapthopathology of ASD.

Nitrosative stress also disrupts GABAergic and glutamatergic signaling in the brain;^[^ ^77^, ^78^ ^]^ the SNO of the NR1^[^ ^79^ ^]^ and GAD65^[^ ^80^ ^]^ lead to their degradation. In agreement with our results, NO donor leads to the downregulation of the GABAergic and glutamatergic markers. The mutant ASD mice with elevated NO levels in the brain also showed similar results.^[^ ^36^ ^]^ Administering 7‐NI for both mutant models and in iPSC‐derived cortical neurons from patients with Shank3 mutations restored the glutamatergic and GABAergic markers. This indicates that NO plays a role in disrupting the glutamatergic and GABAergic systems in ASD. Behavioral studies revealed that nitrosative stress induces behavioral abnormalities in rodents.^[^ ^81^, ^82^ ^]^ Similar to both the Shank3^[^ ^17^, ^83^, ^84^ ^]^ and Cntnap2^(‐/‐)[^ ^22^, ^25^, ^85^ ^]^ mouse models, WT mice treated with NO donor exhibited a reduced interest in novel objects, as well as impaired sociability and increased anxiety. However, administering 7‐NI led to a reversal in most of the behavioral phenotypes in mutant mice. NO donor administration in mutant mice worsens some of the behavioral phenotypes in both male and female mice. These results suggest that high NO concentrations in ASD pathology could potentially induce behavioral and cognitive deficits. Collectively, we concluded that inhibition of NO production leads to the reversal of ASD‐like behavior in mutant mice.

In addition, we selectively cultured primary cortical neurons from both mutant groups to rule out the effects of non‐neuronal cell types in NO production due to *SHANK3* and *Cntnap2* mutations. Thus, like the in vivo results, we found a loss of Syp expression and elevated levels of 3‐Ntyr in the neuronal cells. Treating these cells with nNOS inhibitor significantly restored these parameters. This result confirms the involvement of nNOS in aberrant NO production, leading to molecular and cellular ASD deficits. Similarly, genetic manipulation of nNOS in *SHANK3* knocked‐down human cell lines showed a reversal in 3‐Ntyr and synaptic defects. Collectively, our targeted pharmacological and genetic manipulations of nNOS showed its involvement in ASD.

Bioinformatics analysis of the SNO‐proteome of human blood samples revealed an enrichment of key BPs that are associated with ASD progressions, such as regulation of MAPK cascades,^[^ ^86^ ^]^ nervous system diseases,^[^ ^87^ ^]^ a complement system in neuronal development and plasticity,^[^ ^88^ ^]^ and oxidative damage response,^[^ ^89^ ^]^ which is consistent with the above references. GO classification analysis of the SNO‐proteins showed the involvement of complement system proteins in ASD plasma samples. Previous studies have demonstrated the involvement of complement systems in ASD.^[^ ^90^, ^91^, ^92^ ^]^ Notably, NO plays a role in complement system activation.^[^ ^93^, ^94^ ^]^ However, further investigation is needed to study the role of NO in complement systems in ASD.

Collectively, our results show for the first time that NO plays a key role in ASD development. We found that NO affects synaptogenesis as well as the glutamatergic and GABAergic systems in the cortex and the striatum, which converge into ASD‐like behavioral deficits. This work suggests that NO is an important pathological factor in ASD. Examining NO in diverse mutations on the spectrum as well as other neurodevelopmental disorders and psychiatric diseases will open novel future research directions. Finally, this is a novel experimental study that establishes a direct link between NO and ASD, leading to the discovery of novel NO‐related drug targets for the disorder and suggesting nNOS as a precise target for treatment.

## 4 Experimental Section

### Materials

Primary antibodies, Anti‐GAD1 (#41 318), Anti‐NeuN (#94 403), Anti‐*β*‐Actin (#3700), Anti‐PSD95 (#36 233), Anti‐Caspr2 (#3731), Anti‐nNOS (#4231), Anti‐synaptophysin (#36 406) and secondary antibodies, Anti‐Rabbit Alexa fluor 594 (#8889), Anti‐mouse (Alexa Fluor 488 (#4408), HRP‐conjugated anti‐rabbit (7076S), HRP‐conjugated anti‐mouse (7074S), ProLong gold Antifade with DAPI (#8961), and protease phosphatase inhibitor cocktail (#5872) were purchased from Cell Signaling Technology (Danvers, MA, USA). Anti‐3Ntyr antibody (ab110282), Anti‐homer 1 (ab184955), Anti‐NMDAR1 antibody (ab109182), Anti‐SLC32A1/VGAT antibody (1b235952), and anti‐MAP2 (ab11268) were purchased from Abcam, Cambridge, UK. Anti‐Shank3 (sc‐377088) was purchased from Santa Cruz Biotechnology, Dallas, TX, USA. Other general chemicals were purchased from Sigma Aldrich (St. Louis, MO, USA) and Bio‐Rad Laboratories (Haifa, Israel).

### Animal Housing, Pharmacological Interventions, and Tissue Dissection

All animal experiments were conducted under the guidelines of the Institutional Animal Care and Use Committee and the Association for Assessment and Accreditation of Laboratory Animal Care International (IACUC‐ MD‐20‐16049‐3). Shank3^Δ4‐22^ (Strain #:03 2169), Cntnap2^(‐/‐)^ (Strain #:01 7482), and C57BL/6J (Strain #:000664) were purchased from the Jackson Laboratory (Farmington, CT, USA). The Shank3^Δex4‐22^ mice strain is characterized by the deletion of exons 4–22 of the gene encoding the SH3 and multiple ankyrin repeat domains 3 (Shank3). We mated hetero‐male Shank3^Δex4‐22^ mice and hetero‐female Shank3^Δex4‐22^ mice. We obtained 3 genotypes: WT, hetero, and homo Shank3^Δex4‐22^ mice. Here we used WT littermates as a control for the Shank3 experiments. For Cntnap2^(‐/‐)^ we used homozygous mating pairs. C57BL/6J is used as a control group for Cntnap2^(‐/‐)^. Male Shank3^Δex4‐22[^ ^95^ ^]^ and their control littermates, as well as Cntnap2^(‐/‐)^(25), and Wild‐Type (WT) C57BL/6 mice were used in this study for the biochemistry and behavioral assays. The animals were kept at a room temperature (RT) of 23 °C in a 12‐h light/dark cycle and fed ad libitum with standard mouse chow and water.

The pharmacological interventions were performed by daily intraperitoneal (IP) injection. An NO donor, SNAP, was administered to the WT and Shank3^Δ4‐22^ mutant mouse group at 20 mg kg^−1^.^[^ ^54^ ^]^ A nNOS inhibitor, 7‐NI, was injected into the Shank3^Δ4‐22^ mutant, Cntnap2^(‐/‐)^ group, the Shank3 WT littermates, and C57BL/6J at a dose of 80 mg kg^−1^.^[^ ^96^ ^]^ All WT and mutant groups were also treated with their respective vehicle control. Thus, the following groups of mice were used in this study:^[^ ^1^ ^]^ WT (C57BL/6J treated with vehicle),^[^ ^2^ ^]^ WT+SNAP (C57BL/6J mice treated with SNAP),^[^ ^3^ ^]^ WT1 (Shank3 WT treated with vehicle),^[^ ^4^ ^]^ M1(Shank3^Δ4‐22^ mice treated with vehicle),^[^ ^5^ ^]^ M1+7‐NI (Shank3^Δ4‐22^ mice treated with 7‐NI),^[^ ^6^ ^]^ WT2 (C57BL/6J mice treated with vehicle),^[^ ^7^ ^]^ M2 (Cntnap2^(‐/‐)^ mice treated with vehicle),^[^ ^8^ ^]^ M2+7‐NI (Cntnap2^(‐/‐)^ mice treated 7‐NI),^[^ ^9^ ^]^ WT1 +7‐NI (Shank3 WT treated with 7‐NI),^[^ ^10^ ^]^ WT2 +7‐NI (C57BL/6J mice treated with 7‐NI),^[^ ^11^ ^]^ M1+SNAP (Shank3^Δ4‐22^ mice treated with SNAP), and^[^ ^12^ ^]^ M2+SNAP(Cntnap2^(‐/‐)^ mice treated with SNAP). Both male and female mice were used in this study. After finishing the treatment period, the animals were euthanized for further experiments. For western blot analysis, the entire cortex and the striatum were isolated, snap‐frozen in liquid nitrogen, and stored at −80 °C for further use, as described previously.^[^ ^36^ ^]^ For IF, mice were intracardially perfused with 0.9% saline and 4% paraformaldehyde. The whole brain was isolated and stored in 10% paraformaldehyde for 24 h. Thereafter, the brains were gradually dehydrated with 10%, 20%, and 30% sucrose gradients. Cryostat (Leica company, Wetzlar, Germany) was used to cut the brain sections.

Sample sizes were chosen based on our preliminary experiments and the researchers' expertise in conducting similar experiments. The experimental design employed a blind‐coding approach in which the data collectors were masked to the genotype of the mice to minimize bias in the data collection process.

### Western Blots

Tissues were homogenized in freshly prepared RIPA buffer (30 mm HEPES, pH 7.4, 150 mm NaCl, 1% Nonidet P‐40, 0.5% sodium deoxycholate, 0.1% sodium dodecyl sulfate, 5 mm EDTA, 1 mm NaV04, 50 mm NaF, 1 mm PMSF, 1% protease, and phosphatase inhibitors cocktail, pH 7.7) on ice using a Teflon pestle and a Jumbo Stirrer (Thermo Fisher Scientific, Waltham, MA, USA). The homogenates were centrifuged at 17 000 × *g* for 30 min at 4 °C. The supernatant was collected, and the protein concentration was estimated by Bicinchoninic Acid (BCA) Protein Assay (Sigma Aldrich). The samples were subjected to polyacrylamide gel electrophoresis, followed by wet transfer onto a polyvinylidene fluoride (PVDF) membrane (Bio‐Rad Laboratories). Non‐specific sites were blocked by either 5% dried skimmed milk or 5% bovine serum albumin (BSA) in Tris‐buffered saline with Tween 20 (TBST), containing 135 mm NaCl, 50 mm Tris, and 0.1% Tween 20, pH 7.4, for 2 h at room temperature. PVDF membranes containing transferred proteins were incubated with a primary antibody overnight at 4 °C under shaking conditions. The following primary antibodies were used: anti‐Syp (1:1000), anti‐Homer (1:1000), anti‐NR1 (1:1000), anti‐GAD1 (1:1000), anti‐nNOS (1:1000), anti‐PSD95 (1:1000), anti‐iNOS (1:1000), anti‐VGAT (1:1000), anti‐*β*‐Actin (1:1000), anti‐3‐Ntyr (1:1000), and anti‐Shank3 (1:1000). After exposure to primary antibodies, the membranes were washed with TBST and incubated with anti‐mouse/rabbit HRP secondary antibody for 1 h at room temperature. Specific binding of the protein of interest was detected using ECL substrate (Bio‐Rad Laboratories). The bands were visualized using the Bio‐Rad Chemidoc imaging system (Hercules, CA, USA). For quantification, we analyzed the gray value of the protein by using Bio‐Rad Image Lab software; we normalized it to that of the corresponding internal control beta‐actin.

### Nitrite Assay

The detection of nitric oxide metabolites was measured with a Griess assay kit (Sigma‐Aldrich, Cat. No. 23 479) using the manufacturer's instructions.^[^ ^97^ ^]^ A fresh brain was used for this assay. The homogenate was prepared in PBS and filtered with a 10WCO centrifuge tube. An equal amount of sample was assessed for the nitrite level following the manufacturer's protocol. The absorbance was measured at 540 nm.

### Cell Cultures

Human neuroblastoma (SH‐SY5Y) cells were obtained from the American Type Culture Collection (Manassas, VA, USA) and maintained in a 1:1 mixture of Ham's F‐12 and Dulbecco's modified Eagle's medium (DMEM) supplemented with 10% fetal bovine serum, 2 mm l‐glutamine, and 1% penicillin‑streptomycin in a humidified atmosphere at 37°C and 5% CO_2._ Cells were plated onto a non‐coated 35 mm dish at a density of 6.0 × 10^4^ cells cm^−2^ for western blotting and immunocytochemistry.

### SH‐SY5Y Differentiation

SH‐SY5Y cell differentiation was carried out by applying Retinoic acid (RA) and BDNF simultaneously. We used DMEM: F‐12, 1%FBS, and 10 µm retinoic acid as a differentiation media. In brief, the cells in passage numbers 6–8 were used in this experiment and kept under differentiation media over a period of 11–12 days. The media were changed every alternate day. Then, the differentiated cells were further used in the experiment.^[^ ^98^ ^]^

### siRNA Transfection

SH‐SY5Y cells were cultured at a density of 6.0 × 10^4^ cells cm^−2^ and transfected with 20 nm of SHANK3 (sequence‐ 5“CGA UGA UAA GCA GUU UGC AAA GCU U 3’) and 25 nm of nNOS siRNA (sequence‐ 5”CCG UGU CCA ACA UGC UCC UAG AGA U 3’) (Life Technologies) simultaneously for 24 h, using Lipofectamine RNAi MAX (#13 778 030, Life Technologies) according to the manufacturer's protocol. Then, the media was changed after 6 h. Finally, after 72 h the cells were washed with PBS and harvested for western blotting, and cells cultured on a coverslip were used for confocal microscopy.^[^ ^99^ ^]^

### Primary Cortical Neuronal Cultures

Primary cortical neurons were isolated from fetal mouse brains on embryonic days 16–17. In brief, the cortex isolated from embryonic mouse brains was placed in DMEM (Thermo Fisher Scientific) and incubated with 0.25% trypsin + 0.02% EDTA (Sigma‐Aldrich) at 37 °C for 15 min. Neurons were mechanically dissociated by pipetting and then seeded on a poly‑d‑lysine‐coated glass (Sigma‑Aldrich) in six‐well plates. The density was 3 × 10^5^ in six wells on 22 mm PDL‐coated slides for microscopic observation. Cells were cultured in neurobasal medium (Gibco) containing 2% B27 supplement, and 1% penicillin‑streptomycin, in a humidified incubator at 37 °C, 95% air, and 5% CO_2_. On the second day of neuronal culture, half the media volume was replaced with the same volume of fresh neurobasal media with 2 µm cytosine‐*β*‐d‐arbinofuranoside (Sigma‐Aldrich) to avoid glial cell proliferation. The half media was changed with new media every 3rd day. Cytosine‐*β*‐d‐arbinofuranoside was treated only until the 7th day, and the cells were grown for 14 days.^[^ ^100^ ^]^

### *SHANK3* iPSC Culture and Differentiation into Cortical Neurons

We differentiated the previously characterized iPSC lines for *Shank3* mutation (C.3679insG)^[^ ^101^ ^]^ and the respective control iPSC line into cortical neural progenitor cells (NPCs) as described previously.^[^ ^102^, ^103^ ^]^ Furthermore, for differentiation into cortical neurons, the cortical NPCs were dissociated with StemPro Accutase (ThermoFisher Scientific, cat# A1110501) cell dissociation reagent; seeded on Poly‐*l*‐ornithine(Merck, cat# P4957)/Laminin (ThermoFisher Scientific, cat# 23 017 015) coated tissue culture plates, and differentiated using the neuronal differentiation media composed of Brainphys neuronal media (Stem cell technologies, cat# 0 5790) with 1X B27 supplement (ThermoFisher Scientific, cat# 17 504 044); 1X N2 supplement (ThermoFisher Scientific, cat# 17 502 048); 0.2 nm Ascorbic Acid (Stem cell technologies, cat# 72 132); 500 µg mL^−1^ cyclic‐AMP (TOCRIS, cat# 1141); 20 ng mL^−1^ BDNF (Peprotech, cat# 450‐02); 20 ng mL^−1^ GDNF (Peprotech, cat# 450‐10), and 1 ug mL^−1^ laminin (ThermoFisher Scientific, cat# 23 017 015) for 4 weeks. For immunocytochemistry (ICC), approximately 150 000–170 000 dissociated NPCs were seeded on Poly‐*l*‐ornithine/Laminin‐coated 13 mm glass coverslips (Fisher Scientific, cat# 10 513 234) in 24‐well tissue culture plates (Corning, cat# 09‐761‐146). The differentiation was started with the above‐mentioned media after 24–36 h when the NPCs were fully attached to the coverslips. Similarly, for western hybridization, NPCs were dissociated with Accutase, and ≈800 000 cells were seeded in Poly‐*l*‐ornithine/Laminin‐coated six‐well tissue culture plates (Corning, cat# 07‐200‐83). The differentiated neurons were collected at 4 weeks following Accutase treatment in six‐well tissue culture plates for western hybridization, whereas, for ICC, we fixed them with 4% paraformaldehyde for 15 min at room temperature, followed by three PBS washes; the immunostaining was done as described previously.^[^ ^104^ ^]^

Additionally, the cortical neurons of the *Shank3* mutant lines were also treated with a 50 µm concentration of 7‐NI for 48 hours for both ICC and western blotting. Before treatment with the 7‐NI drug, the suitable drug concentration was determined by cytotoxicity assay via Trypan blue exclusion assay, as described below.

### Cytotoxicity Assay on *SHANK3* iPSC‐Derived Cortical Neurons after 7‐NI Drug Treatment

The cytotoxicity assay was performed using a Trypan blue exclusion assay with a Bio‐Rad TC20 automated cell counter following the manufacturer's protocol. Briefly, *Shank3* mutant cortical NPCs were dissociated with Accutase and ≈70 000–80 000 cells were seeded in each well of Poly‐*l*‐ornithine/Laminin‐coated 48‐well tissue culture plates (Corning, cat# 07‐200‐86) a day before differentiation was started with the above‐mentioned neuronal differentiation media. After cortical differentiation for three weeks, the 7‐NI drug was added at a concentration of 10, 20, 35, 50, 75, and 100 micromolar concentrations for 48 h. Next, the cells were collected following Accutase treatment, diluted (1:10) in 100 µL DPBS and further diluted (1:2) with 10 µL, 0.4% Trypan blue dye (Bio‐Rad, cat# 1 450 022). The cells were then loaded onto Bio‐Rad dual‐chambered cell counting slides (Bio‐Rad, cat# 140 003), and the live cell readings were taken on the TC20 automated cell counter. The experiment was conducted with six replicates for each concentration. We found the 50‐µm concentration to have the same cytotoxicity as the lower concentrations of the drug (10,20, and 35 µm).

### cGMP and cAMP Activity Assay

The assay was performed in the 10% homogenate of a cortex sample. WT (WT1 and WT2), mutant (M1 and M2), and mutant + 7‐NI were the groups used in this assay. Samples were prepared in the lysis buffer and assayed as stated in the manufacturer's manual from Cell Signaling (Cell Signaling, Danvers, MA, USA). The absorbance was taken at 450 nm.

### MTT Cell Viability Assay

The effects of 7‐NI on primary cortical neurons were determined using a colorimetric 3‐(4,5‐dimethylthiazol‐2‐yl)‐2,5‐diphenyl‐tetrazolium (MTT) assay (Sigma‐Aldrich). Five or six duplicates of each treatment were performed in each experiment.

### Golgi Staining Protocol and Spine Density Measurement

Golgi staining and spine counting were performed as described previously.^[^ ^105^ ^]^ Nine littermate pairs of male juvenile mice were used for this experiment. Golgi staining of the mouse brain was performed as instructed in the user manual for the FD Rapid GolgiStain Kit (FD NeuroTechnologies, Columbia, MD, USA). In brief, first, the mice were intracardially perfused with 0.9% saline. Next, the brain was dissected. The dissected brains were first immersed in the impregnation solution (solution A+B) for 21 days in the dark, then incubated in solution C for 3 days before slicing. A vibratome was used to cut 100 µm‐thick coronal sections of mouse brains. After slide preparation, the sections were incubated in staining solutions (Solution D/E) for 10 min and washed twice with distilled water after staining. The samples were then dehydrated by sequential rinsing in 50%, 75%, 95%, and 100% ethanol, cleared with xylenes, and mounted with a mounting medium. A Nikon‐TL confocal microscope was used to capture the images. The study included cells with their cell body located in the somatosensory cortex of mouse brains and with complete or nearly complete dendritic arbors. Cells with poor impregnation were excluded. Each cell was observed under a microscope at 100× magnification and drawn, with the total length of its dendrites measured. Subsequently, at 1000× magnification, spines were counted on all dendrites that protruded laterally from the shafts of the dendrites into the surrounding neuropil. Only those spines were included in the spine count to ensure consistency. The spine density of a pyramidal neuron was calculated by dividing the total number of spines on the neuron by the total length of its dendrites, and expressed as the number of spines per unit length (i.e., spines/50 um). Finally, the mean spine density for each group was calculated. The density of dendritic spines was analyzed using Image J software. All imaging and analysis were conducted in a blind fashion.

### Confocal Microscopy for Mouse Brain Sections

Coronal sections of the cortical region (20 µm thick) were processed for dual immunofluorescence. In brief, the sections were incubated in blocking buffer, followed by mouse anti‐NeuN (1:500), rabbit anti‐NR1 (1:200), anti‐mouse 3‐Ntyr (1:200), and rabbit anti‐GAD1 antibodies (each diluted 1:200). Then, the sections were rinsed with PBS and incubated with anti‐rabbit Alexa fluor 594 (1:1000) and anti‐mouse Alexa Fluor 488 (1:1000) secondary antibodies for 2 h in the dark. After secondary incubation, the sections were washed with PBS 3 times and mounted on glass slides with DAPI. Confocal images were captured using 40×/60×/100× with a Nikon confocal microscope and a sequential acquisition setting of 2024 × 1024. Each image was a z‐series projection of 8–10 images. The depth intervals of each image were determined by instruments automatically. The mean fluorescence intensity was calculated using Image J software, FUJI, and JACoP plugin (NIH, Bethesda,MD) as described elsewhere.^[^ ^106^ ^]^

### Confocal Microscopy for Cultured Cells

Cells cultured on coverslips were washed three times with PBS and fixed with 4% paraformaldehyde for 15 min at room temperature. The fixed cells were washed several times with PBS and incubated in the absence or presence of permeabilization buffer (PBS containing 0.1% Triton X‐100) for 5 min at room temperature. After washing the cells three times with PBS, they were blocked with blocking buffer (PBS containing 5% BSA) for 30 min at room temperature and then incubated with the primary antibodies overnight at 4 °C. Next, the samples were incubated with anti‐rabbit Alexa fluor 594 (1:1000) and anti‐mouse (Alexa Fluor 488 (1:1000) secondary antibodies for 2 h, washed with PBS, and mounted with the nucleus fluorescent probe DAPI. Finally, the cells were observed (65×) under a Nikon confocal microscope.

### Human Participants

All experimental protocols were approved by the Shaare Zedek Medical Center Institutional Review Board (Jerusalem, Israel; IRB# 0501‐20‐SZMC, original approval 02 December 2020). Whole blood samples were collected from 19 children with ASD and 20 unrelated, age‐, and gender‐matched, neurotypical children who attended regular education classes and did not have any neuropsychiatric diagnosis. The demographic and symptom severity data of the cohorts are presented in Table S2, Supporting Information. Participants were recruited from the outpatient clinics and through advertisements posted in the surrounding community of Jerusalem, Israel. The study and its methods were performed in accordance with approved protocols and relevant guidelines and regulations. Informed consent was obtained.

### Whole Blood Sample Collection

Venous whole blood was collected in the morning hours from non‐fasting individuals. Whole blood samples were centrifuged within 15 min from the blood draw, and the plasma aliquots were frozen immediately at −80 °C until use.

Lysis buffer was added to plasma and kept at 4 °C for 10 min. Samples were centrifuged for 12 min at 4 °C. The supernatant was collected, and 0.5 to 1% of SDS was added. Supernatant samples were washed with 50 mm HEPES buffer and centrifuged at 5000 × *g* for 30 min at 10 °C. Then 1.5 mm of SNOTRAP was added to the sample and incubated for 2 h at room temperature (32, 61). Samples were washed with 50 mm ammonium bi carbonate (ABC) and centrifuged at 5000 × *g* for 25 min at 10 °C. Washed samples were mixed with streptavidin beads for 2 h at RT and centrifuged at 4 °C. SNO‐protein were washed from beads with ABC buffer. SNO‐proteins were eluted from the beads with 10 mm TCEP.

### nanoLC‐MS/MS Analysis

MS analysis was performed using a Q Exactive Plus mass spectrometer (Thermo Fisher Scientific, Waltham, MA USA) coupled online to a nanoflow UHPLC instrument, Ultimate 3000 Dionex (Thermo Fisher Scientific, Waltham, MA USA). Peptides (0.45 µg, as estimated by O.D.280 nm) were separated over a non‐linear gradient (0–80% acetonitrile) and run at a flow rate of 0.3 µL min^−1^ on a reverse phase 25‐cm‐long C18 column (75 µm ID, 2 µm, 100Å, Thermo PepMapRSLC) for 120 min. The survey scans (380–2000 m/z, target value 3E6 charges, maximum ion injection times 50 ms) were acquired and followed by higher energy collisional dissociation (HCD)‐based fragmentation (normalized collision energy, 25%). A resolution of 70 000 was used for the survey scans, and up to the 15 dynamically chosen most abundant precursor ions, with “a peptide preferred” profile were fragmented (the isolation window was 1.6 m/z). The MS/MS scans were acquired at a resolution of 17 500 (the target value was 1E5 charges; the maximum ion injection times were 120 ms). Dynamic exclusion was 60 s. Data were acquired using Xcalibur software (Thermo Scientific). To avoid a carryover, the column was washed with 80% acetonitrile and 0.1% formic acid for 25 min between samples.

### MS Data Analysis

Mass spectra data were processed using the MaxQuant computational platform, version 2.0.3.0. Peak lists were searched against the translated coding sequences of the human proteome obtained from Uniprot. The search included cysteine carbamidomethylation as a fixed modification and oxidation of methionine as variable modifications; it allowed up to two miscleavages. The match between runs option was used. Peptides with a length of at least seven amino acids were considered, and the required FDR was set to 1% at the peptide and protein levels. Relative protein quantification in MaxQuant was performed using the label‐free quantification (LFQ) algorithm.

### Behavioral Tests

All behavioral experiments were recorded and data were scored automatically through the video tracking software Ethovision XT 16 (Noldus Information technology BV) using AI‐assisted tracking, which tracks mice across two dimensions (*x,y*) while recording three body points (tail, center, and nose). Analysis for the interaction duration tests of the mice used the nose‐point tracking data, and analysis. Our testing setup in the program was consistent and the same template settings were used for the behavioral tests of all mice. The arena was cleaned with 5% virusol between each trial.

### Open Field Test

The motor activity of mice was tested in an open field consisting of a white plastic arena (60 cm × 60 cm). In the first session (habituation), mice were placed individually in the center of the arena and allowed to move freely for 5 min. The next day, the mice were placed in the same field. The total distance traveled (cm) was measured using the Ethovision XT system. After each test, the arena was cleaned with 70% alcohol/virusol solution.^[^ ^58^ ^]^

### Object Recognition Test

To evaluate cognition, particularly recognition memory, two object recognition tests or a novel object recognition test (NOR) were tested. This test utilizes the tendency of mice to explore novel stimuli.^[^ ^107^ ^]^ The test consisted of a familiarization session and a test session. At 24 h before these sessions began, the mice were allowed to explore the arena without objects for 5 min to habituate to their surroundings. During the first 5 min session (the first day), the mice were left to explore two identical objects that could be found at constant locations, 15 cm from the sidewalls, in the already familiar white plastic arena. Twenty‐four hours later, the mice were introduced to the arena for a test session in which one of the familiar objects was replaced by a novel object. Exploration was defined as mice directing their nose to the object at ≤1 cm and/or touching the object with their nose. The time spent by the mouse exploring each object was recorded for 5 min. The arena and the objects were cleaned with 70% alcohol after each session.

### Elevated Plus Maze Test

The elevated plus‐maze^[^ ^108^ ^]^ consisted of four arms (30 × 5 cm): two open and two closed. This test was used to assess the anxiety‐related behavior in rodents. The platform was made of white plexiglass. The apparatus was elevated 45 cm above the floor. The test was initiated by placing the mouse on the central platform of the maze, facing one of the open arms, and letting it move freely. Each session lasted 10 min. The time spent in the closed and open arms was recorded.

### Three‐Chambered Social Test

A three‐chamber sociability test was used to evaluate sociability and novelty‐seeking behavior. Generally, the mice prefer to spend more time with another mouse (sociability), and the mice tend to investigate a novel mouse more than a familiar mouse (social novelty). The social test apparatus consisted of a transparent acrylic box divided into three chambers.^[^ ^109^ ^]^ Two cylindrical wire cages were placed in chambers 1 and 3. On the first day, the test animal was introduced to the middle chamber and allowed to adjust for 5 min for habituation. On the second day, an unfamiliar mouse (age and sex matched) was introduced into a wire cage in one of the side chambers, and the other side chamber remained empty. The time that the test mouse spent exploring the wire cage with an unfamiliar mouse was recorded for 10 min (the sociability test). On the third day, another stranger mouse was introduced into a wire cage in one of the side chambers, and the other side chamber had a familiar mouse. The time spent exploring both the familiar and unfamiliar mice was recorded for 10 min (the social memory test).

### Statistics and Bioinformatics

The mean and Standard Error of the mean (SEM) were calculated for the western blots, immunofluorescence, spine density counting, and behavioral experiments. The discrimination index was also calculated for the behavioral tests. A one‐way ANOVA or a two‐way ANOVA test, along with the Bonferroni multiple comparison test was used for the behavioral test and the western blots. Statistical details and methods used in each experiment can be found in the figure legends. For Biological Processes (BP) analysis, we uploaded the lists of all SNO proteins into STRING, version 11.5. Protein‐protein interaction of SNO‐proteins was also done by STRING (http://string‐db.org).^[^ ^110^ ^]^ High‐confidence interactions (score > 0.7) from the neighborhood, gene fusion, co‐occurrence, co‐expression, experiments, databases, and text‐mining lists were used. The quantification was based on the ion intensity of the peptides. We used Benjamini‐Hochberg correction^[^ ^111^ ^]^ on the p‐value to generate an FDR, and processes/terms with FDR values below 0.05 were included. Bar graphs and statistical analysis were performed using Prism 9.3 (GraphPad Software, San Diego, CA). The data are presented as the mean ± SD. *p < 0.05, **p < 0.001, ***p < 0.0001, and ns = non‐significant.

## Conflict of Interest

The authors declare no conflict of interest.

## Supporting information

Supporting Information

Supplemental Table 1

Supplemental Table 2

Supplemental Table 3

Supplemental Table 4

## Data Availability

The data that support the findings of this study are available on request from the corresponding author. The data are not publicly available due to privacy or ethical restrictions.
