# Supplementary material for: The NO Answer for Autism Spectrum Disorder
Source: Adv Sci (Weinh). 2023 May 22;10(22):2205783. doi: 10.1002/advs.202205783 (PMC10401098; doi:10.1002/advs.202205783)
Supplement: Supplementary file 1 — Supporting Information [file ADVS-10-2205783-s001.pdf]

ADVANCED SCIENCE
Open Access

Supporting Information

for *Adv. Sci.*, DOI 10.1002/advs.202205783

The NO Answer for Autism Spectrum Disorder

*Manish Kumar Tripathi, Shashank Kumar Ojha, Maryam Kartawy, Wajeha Hamoudi, Ashwani Choudhary, Shani Stern, Adi Aran and Haitham Amal**

Supp. Information

The NO Answer for Autism Spectrum Disorder

Manish Kumar Tripathi[1], Shashank Kumar Ojha[1], Maryam Kartawy[1], Wajeha Hamoudi[1], Ashwani Choudhary[2], Shani Stern[2], Adi Aran[3,4], and Haitham Amal[1,#]

[1]Institute for Drug Research, School of Pharmacy, Faculty of Medicine, The Hebrew University of Jerusalem, Jerusalem, Israel

[2]Sagol department of neurobiology, Faculty of natural sciences, University of Haifa, Haifa

[3]Neuropediatric Unit, Shaare Zedek Medical Center, Jerusalem, Israel

[4] Faculty of Medicine, The Hebrew University of Jerusalem, Jerusalem, Israel

# Corresponding Author: Haitham Amal, PhD. Email: haitham.amal@mail.huji.ac.il

Supplementary tables are uploaded as Excel/Word files:

Table 1: MTT assay of 7-NI in primary cortical neuronal culture.

Table 2: Clinical characteristics of the participants (TD and ASD).

Table 3: 3- Nitrotyrosine relative value in plasma TD and ASD.

Table 4: System biology analysis of the plasma samples of TD and ASD children.

Fig. S1.

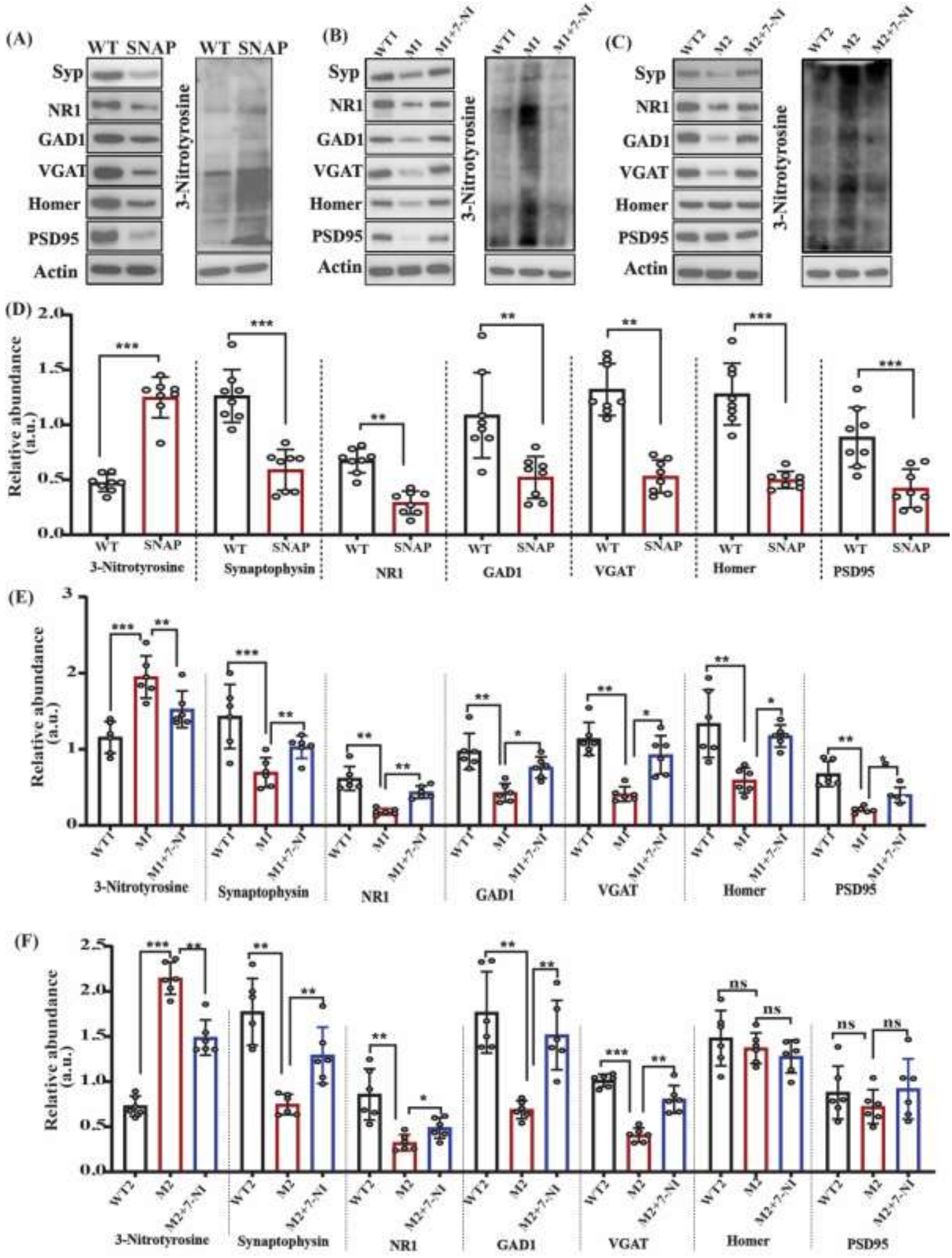

(A)
WT SNAP
Syp
NR1
GAD1
VGAT
Homer
PSD95
Actin
3-Nitrotyrosine
(B)
WT1 M1 M1+7-NI
(C)
WT2 M2 M2+7-NI
(D)
Relative abundance (a.u.)
3-Nitrotyrosine
Synaptophysin
NR1
GAD1
VGAT
Homer
PSD95
(E)
(F)
ns

Fig. S1. Nitric oxide contributes to nitrosative stress and synaptic pathology in the Striatum of *Shank3*$^{\Delta 4\text{-}22}$ mutant (M1), *Cntnap2*$^{(-/-)}$ mutant (M2) mice, and SNAP-treated mice.

Panel A: Representative western blots of an indicator of nitrosative stress 3-Ntyr, and synaptic proteins Syp, NR1, GAD1, VGAT, Homer, and PSD95. β-actin was used as a reference for protein loading. Groups of mice: WT (black6 mice treated with vehicle, n=6); SNAP (WT mice treated with the NO donor compound SNAP, n=6). **Panel B:** Representative western blots of an indicator of nitrosative stress 3-Ntyr, and synaptic proteins Syp, NR1, GAD1, VGAT, Homer, and PSD95. β-actin was used as a reference for protein loading. WT1 (Shank3 WT littermates, n=6), M1 (Shank3 mutant mice, n=6), M1+7-NI (Shank3 mutant treated with the nNOS inhibitor 7-NI, n=6) **Panel C:** Representative western blots of an indicator of nitrosative stress 3-Ntyr, and synaptic proteins Syp, NR1, GAD1, VGAT, Homer, and PSD95. β-actin was used as a reference for protein loading. Groups of mice: WT (n=6); M2 (Cntnap2$^{(-/-)}$ mice treated with vehicle, n=6); and M2+7-NI (M2 mice treated with the nNOS inhibitor 7-NI; n=6). Panel **D**: Statistical analysis of the relative abundance of proteins shown in **Panel A**. **Panel E**: Statistical analysis of the relative abundance of proteins shown in **Panel B**. **Panel F**: Statistical analysis of the relative abundance of proteins shown in **Panel C**. The mean and Standard Deviation (SD) were calculated for all the groups. A one-way ANOVA test with the Bonferroni multiple comparisons tests was used for the western blots analysis. * $p<0.05$, ** $p<0.01$, *** $p<0.001$, ns=non-significant.

Fig. S2.

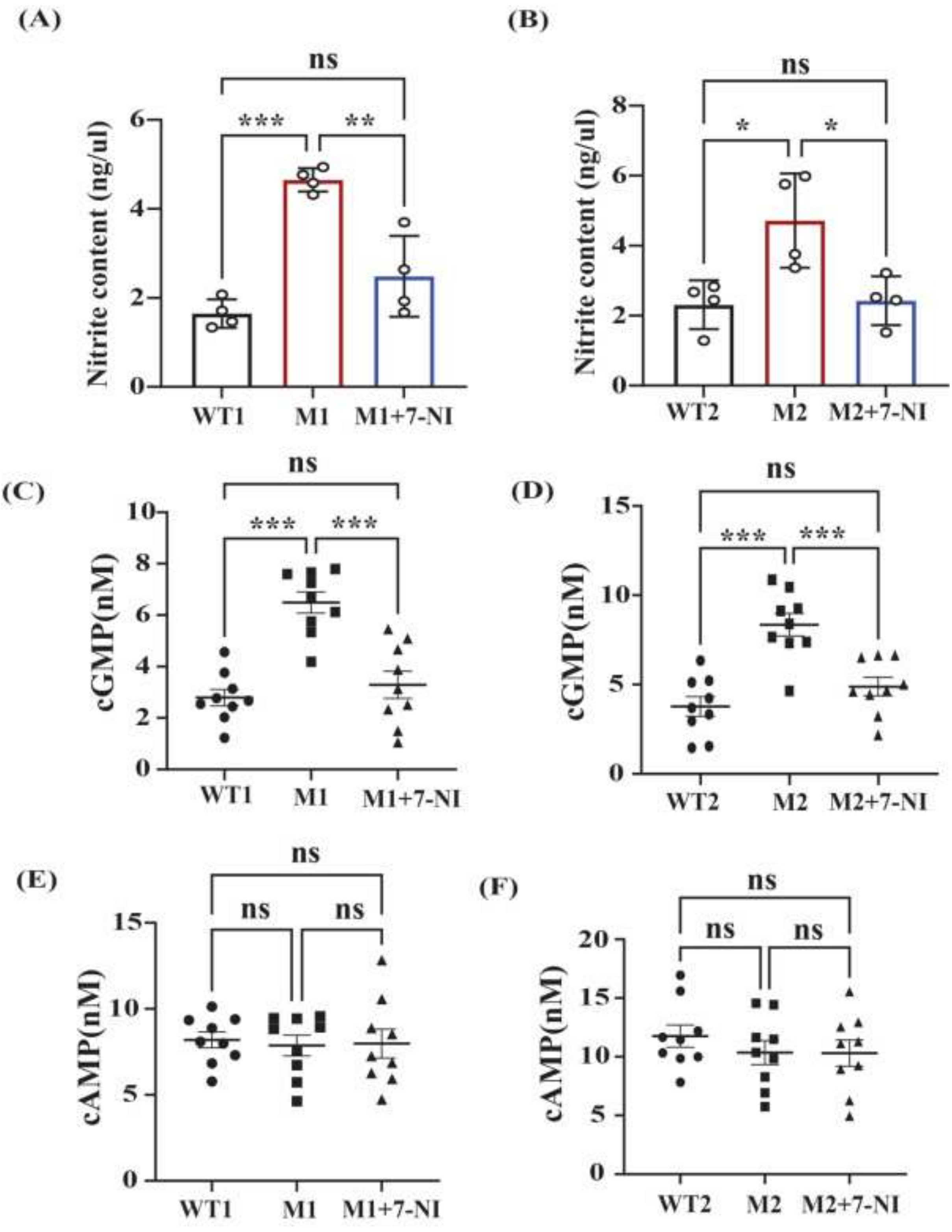

(A)
ns
*** **
Nitrite content (ng/ul)
0 2 4 6
WT1 M1 M1+7-NI
(B)
ns
* *
Nitrite content (ng/ul)
0 2 4 6 8
WT2 M2 M2+7-NI
(C)
ns
*** ***
cGMP(nM)
0 2 4 6 8 10
WT1 M1 M1+7-NI
(D)
ns
*** ***
cGMP(nM)
0 5 10 15
WT2 M2 M2+7-NI
(E)
ns
ns ns
cAMP(nM)
0 5 10 15
WT1 M1 M1+7-NI
(F)
ns
ns ns
cAMP(nM)
0 5 10 15 20
WT2 M2 M2+7-NI

Fig. S2. 7-NI treatment reduces NO metabolites and cGMP production in Shank3 and Cntnap2

Panel A: Statistical analysis of nitrite content in WT1, M1, and M1+7-NI group. **Panel B:** Statistical analysis of nitrite content in WT2, M2, and M2+7-NI group. **Panel C:** Statistical analysis of cGMP level in WT1, M1, and M1+7-NI group. **Panel D:** Statistical analysis of cGMP level in WT2, M2, and M2+7-NI group. **Panel E:** Statistical analysis of cAMP level in WT1, M1, and M1+7-NI group. **Panel F:** Statistical analysis of cAMP level in WT2, M2, and M2+7-NI group. A one-way ANOVA test with the Bonferroni multiple comparisons tests was used for the analysis. * $p<0.05$, ** $p<0.01$, *** $p<0.001$, ns=non-significant.

Fig. S3.

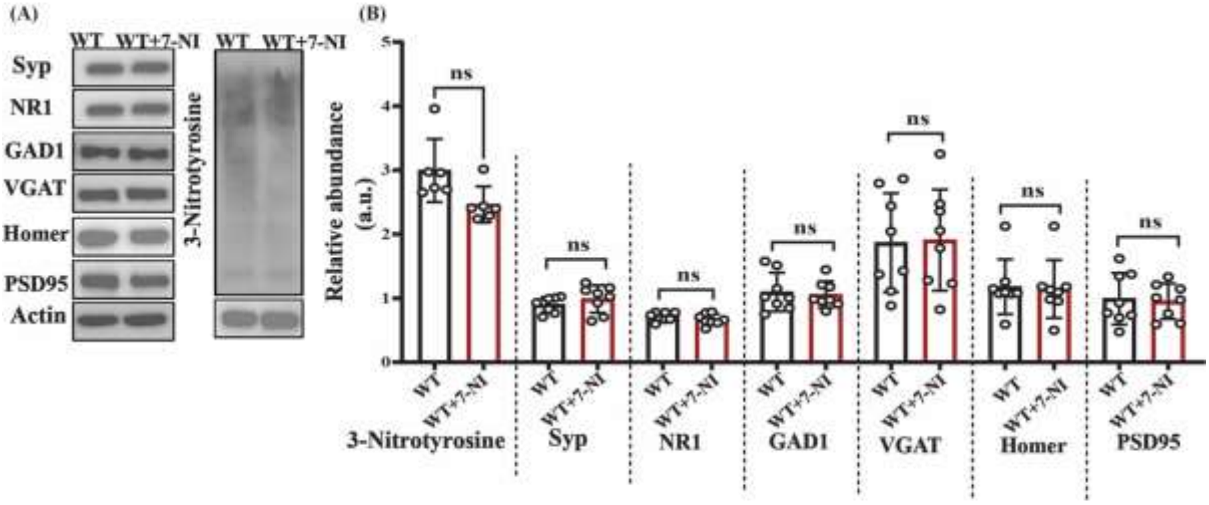

Fig. S3. 7-NI treatment has no effect on the synaptic, glutamatergic, and GABAergic protein levels in the striatum of WT male mice

Panel A: Representative western blots of an indicator of nitrosative stress 3-Ntyr, and synaptic proteins Syp, NR1, GAD1, VGAT, Homer, and PSD95. β-actin was used as a reference for protein loading. Groups of mice: WT (C57BL/6J mice treated with vehicle, n=6); WT+7-NI (WT mice treated with the7-NI, n=6). **Panel B:** Statistical analysis of the relative abundance of proteins shown in **Panel A**. T-test is used for statistical analysis. ns= non-significant.

Fig. S4.

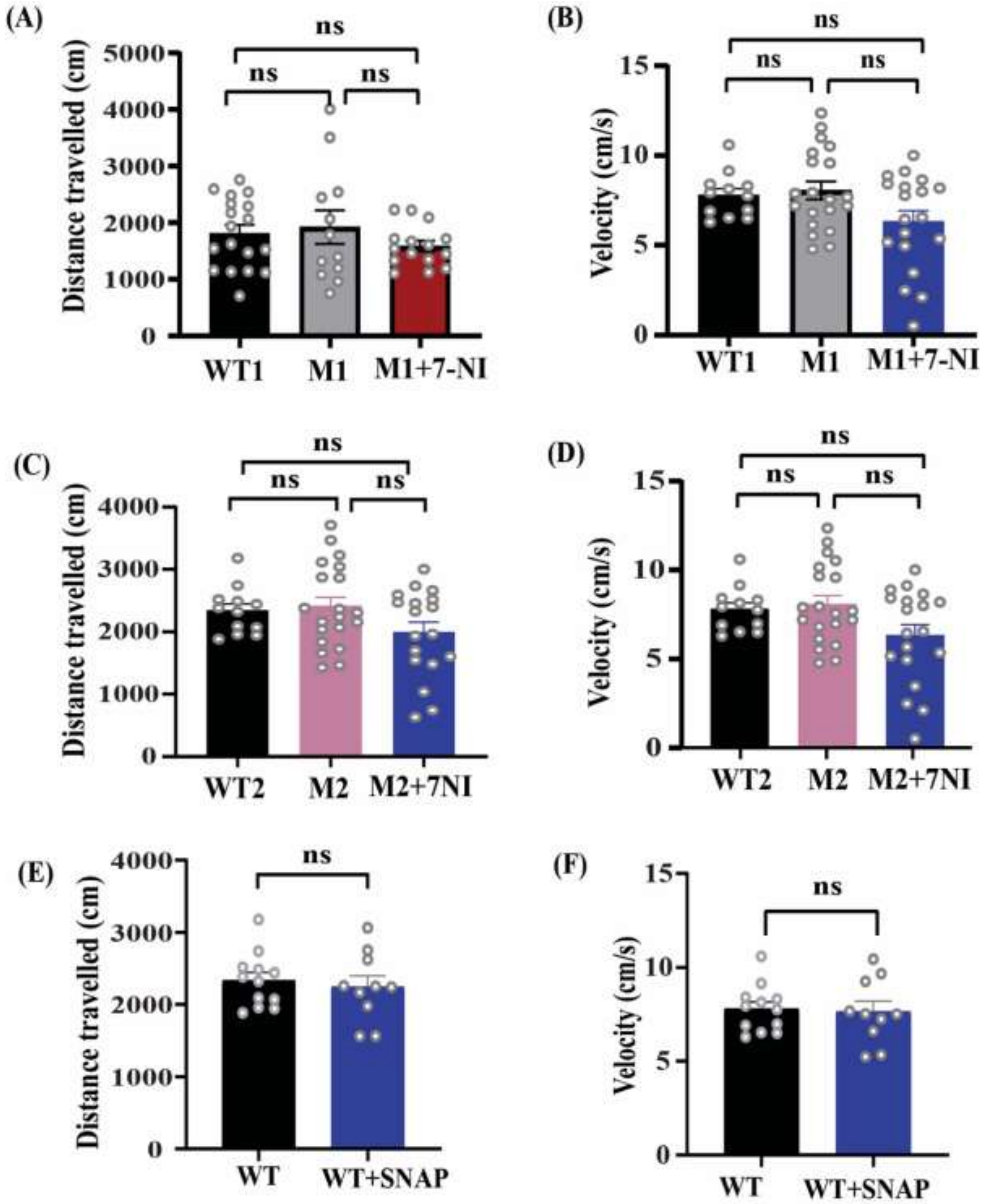
(A)
ns
ns
ns
Distance travelled (cm)
5000
4000
3000
2000
1000
0
WT1
M1
M1+7-NI
(B)
ns
ns
ns
Velocity (cm/s)
15
10
5
0
WT1
M1
M1+7-NI
(C)
ns
ns
ns
Distance travelled (cm)
4000
3000
2000
1000
0
WT2
M2
M2+7NI
(D)
ns
ns
ns
Velocity (cm/s)
15
10
5
0
WT2
M2
M2+7NI
(E)
ns
Distance travelled (cm)
4000
3000
2000
1000
0
WT
WT+SNAP
(F)
ns
Velocity (cm/s)
15
10
5
0
WT
WT+SNAP

Fig. S4. Inhibition of NO production did not affect motor activity in mice

Panel A &B: Open field test- Statistical analysis of the distance traveled and velocity. No significant differences were observed in the distance traveled or velocity among the three male test groups. WT1 (n=18), M1 (n=12), and M1+7-NI (n=15).

Panel C & D: Open field test-Statistical analysis of the distance traveled and velocity. No significant differences were observed in the distance traveled or velocity among the three male test groups. WT2 (n=12), M2 (n=20), and M2+7-NI (n=18).

Panel E & F: Open field test- Statistical analysis of the distance traveled and velocity. No significant differences were observed in the distance traveled or velocity among the male test groups. WT (n=12), and WT+SNAP (n=10). In all tests, the data is presented as mean ± SD. Statistical significance was determined using a Two-way ANOVA with Bonferroni's multiple comparisons tests or two-tailed t-test. ns = non-significant.

Fig. S5.

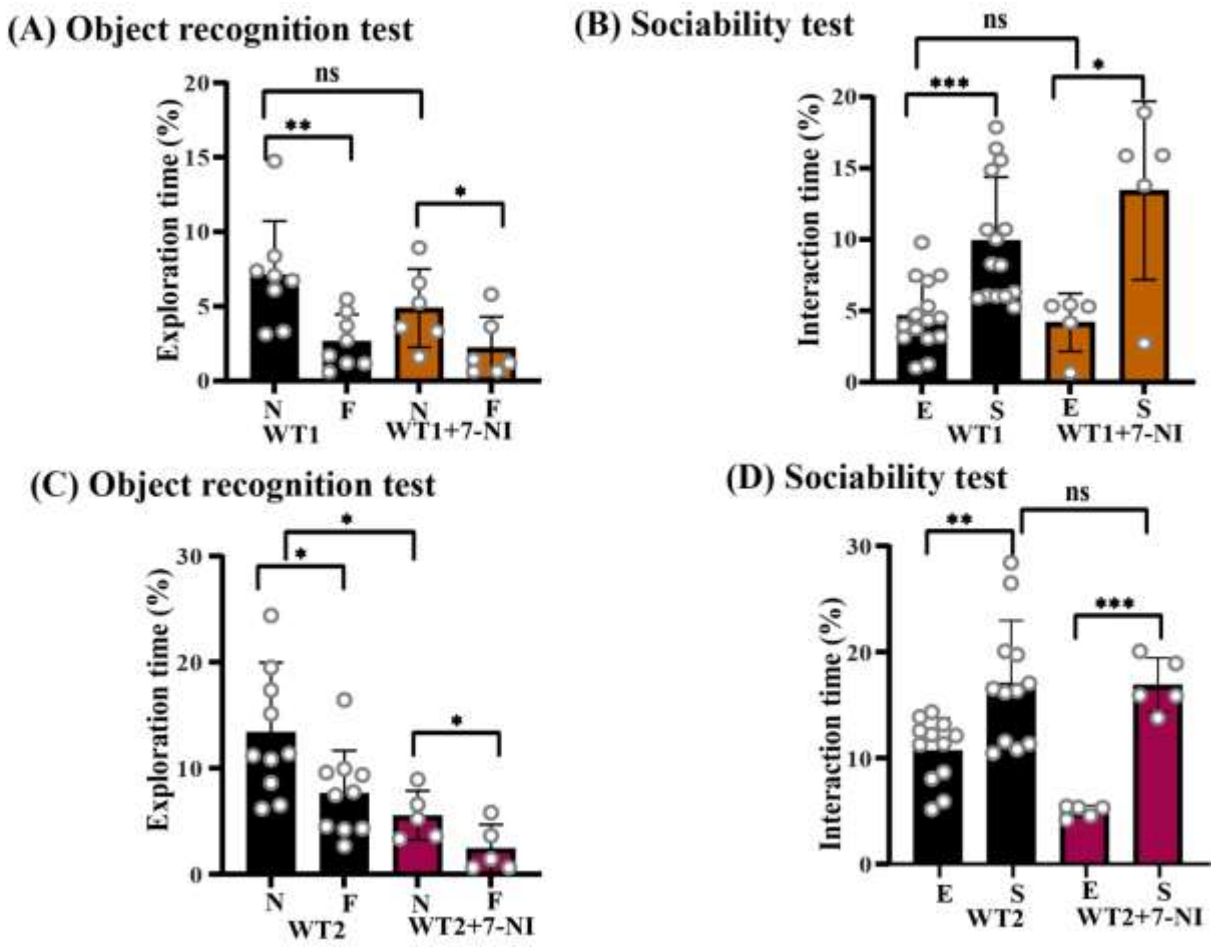
(A) Object recognition test
Exploration time (%)
ns
**
*
N
F
N
F
WT1
WT1+7-NI
(B) Sociability test
Interaction time (%)
ns
***
*
E
S
E
S
WT1
WT1+7-NI
(C) Object recognition test
Exploration time (%)
*
*
*
N
F
N
F
WT2
WT2+7-NI
(D) Sociability test
Interaction time (%)
**
ns
***
E
S
E
S
WT2
WT2+7-NI

Fig. S5. 7-NI treatment has no effect on the WT male mice behavior

Behavioral tests analysis for the following group of male mice: 1. WT1 (Shank3 littermates), 2. WT2 (C57BL/6J), 3. WT1+7-NI (WT1 mice treated with 7-NI), 4. WT2+7-NI (WT2 mice treated with 7-NI) (*i.p.* injection of 80mg/kg).

Panel A: Novel Object recognition test (NOR). The WT1 mice spent significantly more time exploring the novel (N) object than the familiar (F) one (n=8, ***P=0.0074*). WT1+7-NI similarly spent significantly more time exploring the novel object than the familiar object (n=6, **P=0.0397*). **Panel B:** Three-chamber sociability test. Both the WT1 (n=15, ****P*=0.0004) and the WT1+7-NI (n=5, **P*=0.0138) mice spent significantly increased time interacting with the stranger (S) mouse than with the empty (E) cage. **Panel C:** Novel Object recognition test (NOR). The WT2 mice spent significantly more time exploring the novel object than the familiar one (n=10, **P*=0.0146). WT2+7-NI similarly spent significantly more time exploring the novel object than the familiar object (n=6, **P=0.0397*). **Panel D:** Three-chamber sociability test. Both the WT2 (n=12, ***P*=0.0031) and the WT2+7-NI (n=5, ****P*<0.0001) mice spent significantly increased time interacting with the stranger mouse than with the empty cage. In all tests, the data is presented as mean ± SD. Statistical significance was determined using a Two-way ANOVA with Bonferroni's multiple comparisons tests or two-tailed t-test. *P < 0.05, **P < 0.001, ***P < 0.0001, and ns= non-significant.

Fig. S6.

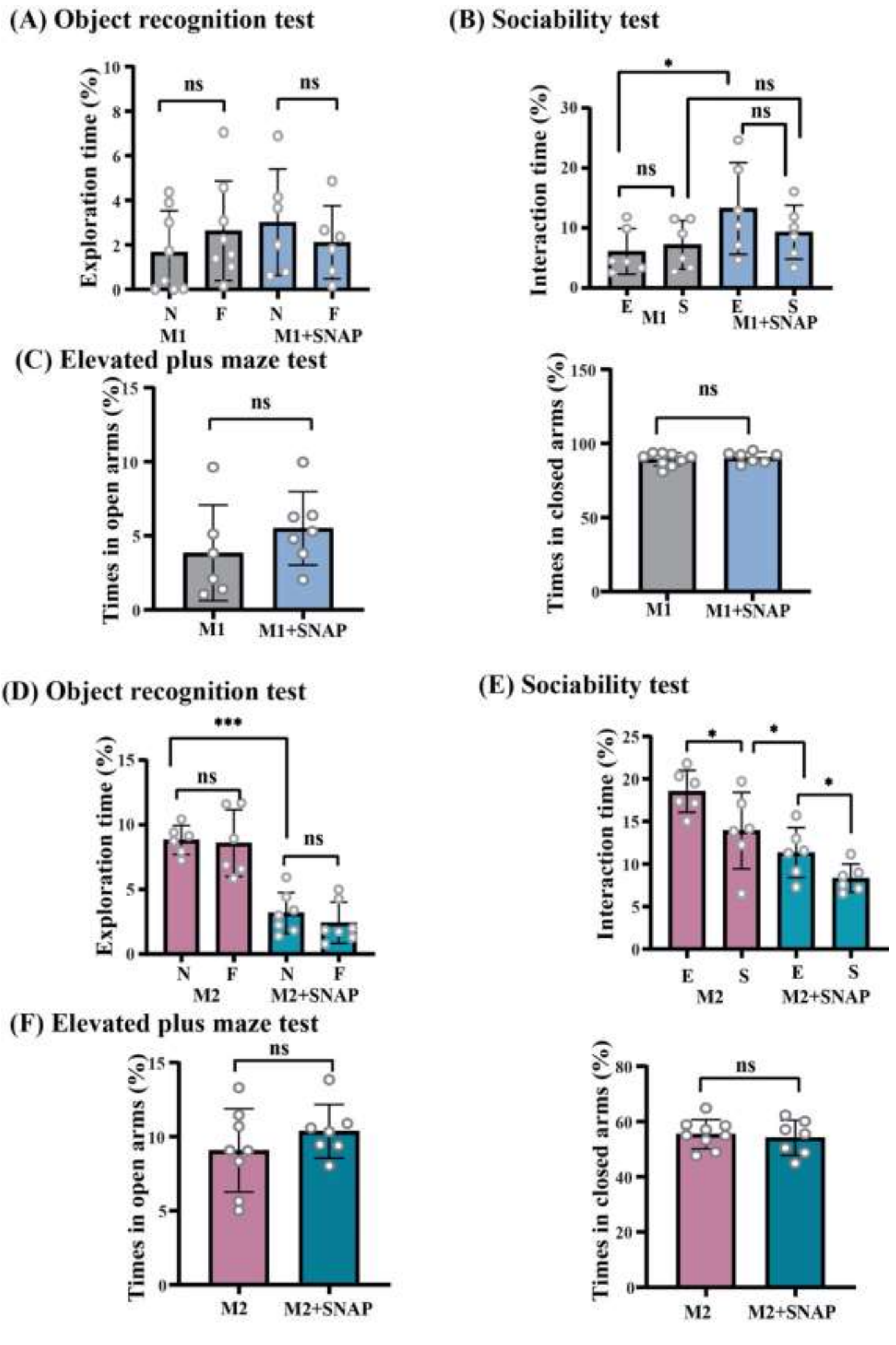
(A) Object recognition test
Exploration time (%)
ns
ns
N
F
N
F
M1
M1+SNAP
(B) Sociability test
Interaction time (%)
*
ns
ns
ns
E
M1
S
E
S
M1+SNAP
(C) Elevated plus maze test
Times in open arms (%)
ns
M1
M1+SNAP
Times in closed arms (%)
ns
M1
M1+SNAP
(D) Object recognition test
Exploration time (%)
***
ns
ns
N
F
N
F
M2
M2+SNAP
(E) Sociability test
Interaction time (%)
*
*
*
E
S
E
S
M2
M2+SNAP
(F) Elevated plus maze test
Times in open arms (%)
ns
M2
M2+SNAP
Times in closed arms (%)
ns
M2
M2+SNAP

Fig. S6. The effect of NO donor on the ASD-like behavior observed in M1 and M2 male mice

Behavioral tests analysis for the following group of male mice: 1. M1 (*Shank3*$^{Δ4-22)}$, 2. M1+SNAP (M1 mice were treated with SNAP), 3. M2 (*Cntnap2*$^{(-/-)}$), 4. M2+SNAP (M2 mice were treated with SNAP) (*i.p.* injection of 20mg/kg).

Panel A: Novel Object recognition test (NOR) showing no significant difference in time exploring the novel (N) object or the familiar (F) one within the M1 (n=8) and M1+SNAP (n=6) male mice. **Panel B:** Both M1 (n=6) and M1+SNAP (n=6) male mice failed to show any significant preference to interact with either the stranger (S) mouse or the empty cage. However, M1+SNAP interacted significantly (*P=0.036) more time with the empty (E) cage compared to the M1 mice. **Panel C:** Elevated plus maze test. No significant difference in time spent in open or closed arms between the M1 (n=6) and M1+SNAP (n=6) was observed. **Panel D:** Novel Object recognition test (NOR). The M2 (n=6) and M2+SNAP (n=6) male mice showed no significant differences in exploring the novel object or the familiar one. However, the M2+SNAP male mice spent significantly (***P<0.0001) less time exploring the novel object compared to the M2 male mice. **Panel E:** Three-chamber sociability test**.** Both the M2 (n=6, *P=0.0262) and the M2+SNAP (n=6, *P=0.0269) mice spent significantly increased time interacting with the empty cage than with the stranger mouse**. Panel F:** Elevated plus maze test. No significant difference in time spent in open or closed arms between the M2 (n=6) and M2+SNAP (n=6) was observed. In all tests, the data is presented as mean ± SD. Statistical significance was determined using a Two-way ANOVA with Bonferroni's multiple comparisons tests or two-tailed t-test. *P < 0.05, **P < 0.001, ***P < 0.0001, and ns=non-significant.

Fig. S7.

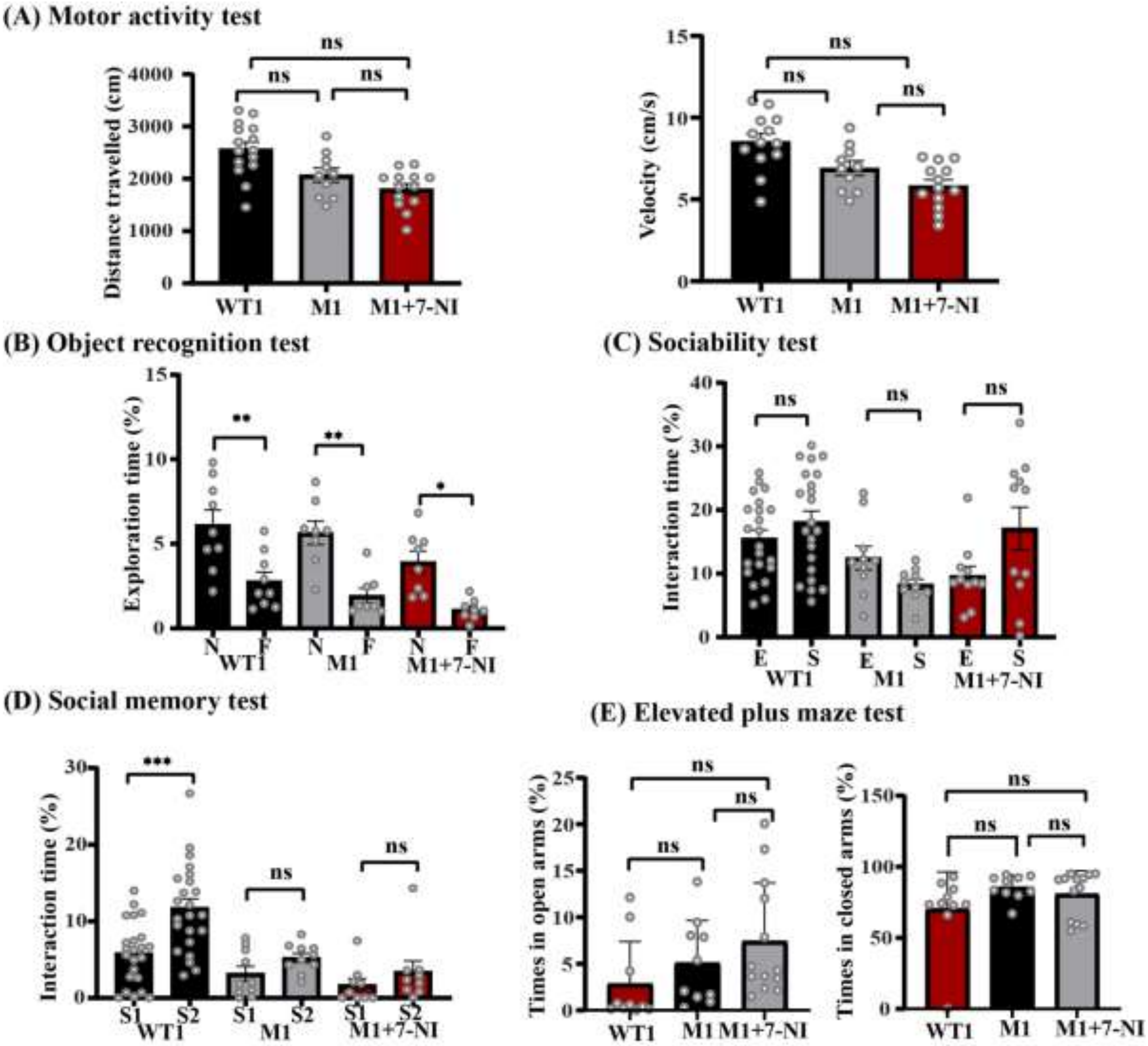

(A) Motor activity test
Distance travelled (cm)
Velocity (cm/s)
WT1
M1
M1+7-NI
ns
(B) Object recognition test
Exploration time (%)
N
F
**
*
(C) Sociability test
Interaction time (%)
E
S
(D) Social memory test
Interaction time (%)
S1
S2
***
(E) Elevated plus maze test
Times in open arms (%)
Times in closed arms (%)

Fig. S7. Testing the ASD-like behavior in the female *Shank3*$^{\Delta 4\text{-}22}$ mouse model.

Panel A: Statistical analysis of the motor activity (distance traveled and velocity) in female WT1 (n=16), M1 (n=10), and M1+7-NI (n=13). **Panel B:** Statistical analysis of the exploration time of a novel (N) and a familiar (F) object among the female mice of WT1 (n=9), M1 (n=8), and M1+7-NI (n=8). **Panel C:** Statistical analysis of time interacting with either an empty (E) cage or stranger (S) mouse among the female mice in WT1 (n=22), M1 (n=10), and M1+7-NI (n=11). **Panel D:** Statistical analysis of time interacting with a familiar or a novel mouse among the female mice in WT1 (n=23), M1 (n=10), and M1+7-NI (n=9). **Panel E:** Statistical analysis of time spent in open or closed arms among the female mice in WT1 (n=10), M1 (n=10), and M1+7-NI (n=13). In all tests, the time is presented as the percentage of the total time. The data is presented as mean ± SD. Statistical significance was determined using a Two-way or One-way ANOVA with Bonferroni's multiple comparisons tests. *$P < 0.05$, **$P < 0.001$, ***$P < 0.0001$, and ns=non significant.

Fig. S8.

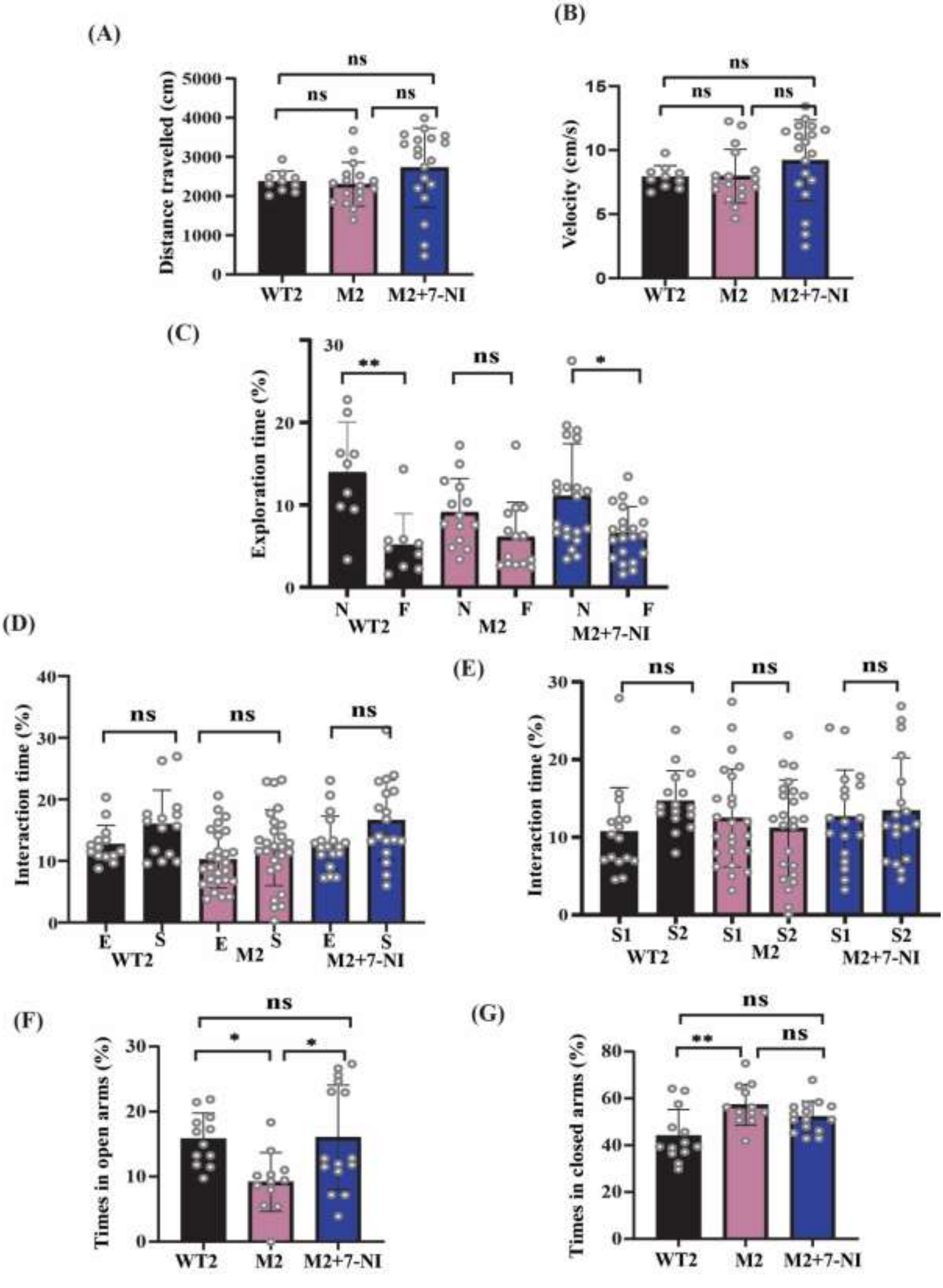
(A)
ns
ns
ns
Distance travelled (cm)
5000
4000
3000
2000
1000
0
WT2
M2
M2+7-NI
(B)
ns
ns
ns
Velocity (cm/s)
15
10
5
0
WT2
M2
M2+7-NI
(C)
Exploration time (%)
30
20
10
0
**
ns
*
N
F
WT2
N
F
M2
N
F
M2+7-NI
(D)
Interaction time (%)
40
30
20
10
0
ns
ns
ns
E
S
WT2
E
M2
S
E
S
M2+7-NI
(E)
Interaction time (%)
30
20
10
0
ns
ns
ns
S1
S2
WT2
S1
S2
M2
S1
S2
M2+7-NI
(F)
Times in open arms (%)
30
20
10
0
ns
*
*
WT2
M2
M2+7-NI
(G)
Times in closed arms (%)
80
60
40
20
0
ns
**
ns
WT2
M2
M2+7-NI

Fig. S8. NO inhibition reversed part of the ASD-like behavior in the female *Cntnap2*$^{(-/-)}$ mouse model.

Panel A & B: Statistical analysis of the motor activity (distance traveled and velocity) in female WT2 (n=10), M2 (n=17), and M2+7-NI (n=19). **Panel C:** Statistical analysis of the exploration time of a novel (N) and a familiar (F) object among the female mice of WT2 (n=9), M2 (n=14), and M2+7-NI (n=21). **Panel D:** Statistical analysis of time interacting with either an empty (E) cage or stranger (S) mouse among the female mice in WT2 (n=14), M2 (n=26), and M2+7-NI (n=18). **Panel E:** Statistical analysis of time interacting with a familiar (S1) or a novel (S2) mouse among the female mice in WT2 (n=17), M2 (n=24), and M2+7-NI (n=18) **Panel F:** Statistical analysis of time spent in open or closed arms among the female mice in WT2 (n=13), M2 (n=12), and M2+7-NI (n=15). In all tests, the time is presented as the percentage of the total time. The data is presented as mean ± SD. Statistical significance was determined using a Two-way or One-way ANOVA with Bonferroni's multiple comparisons tests. *P < 0.05, **P < 0.001, ***P < 0.0001, and ns=non-significant.

Fig S9.

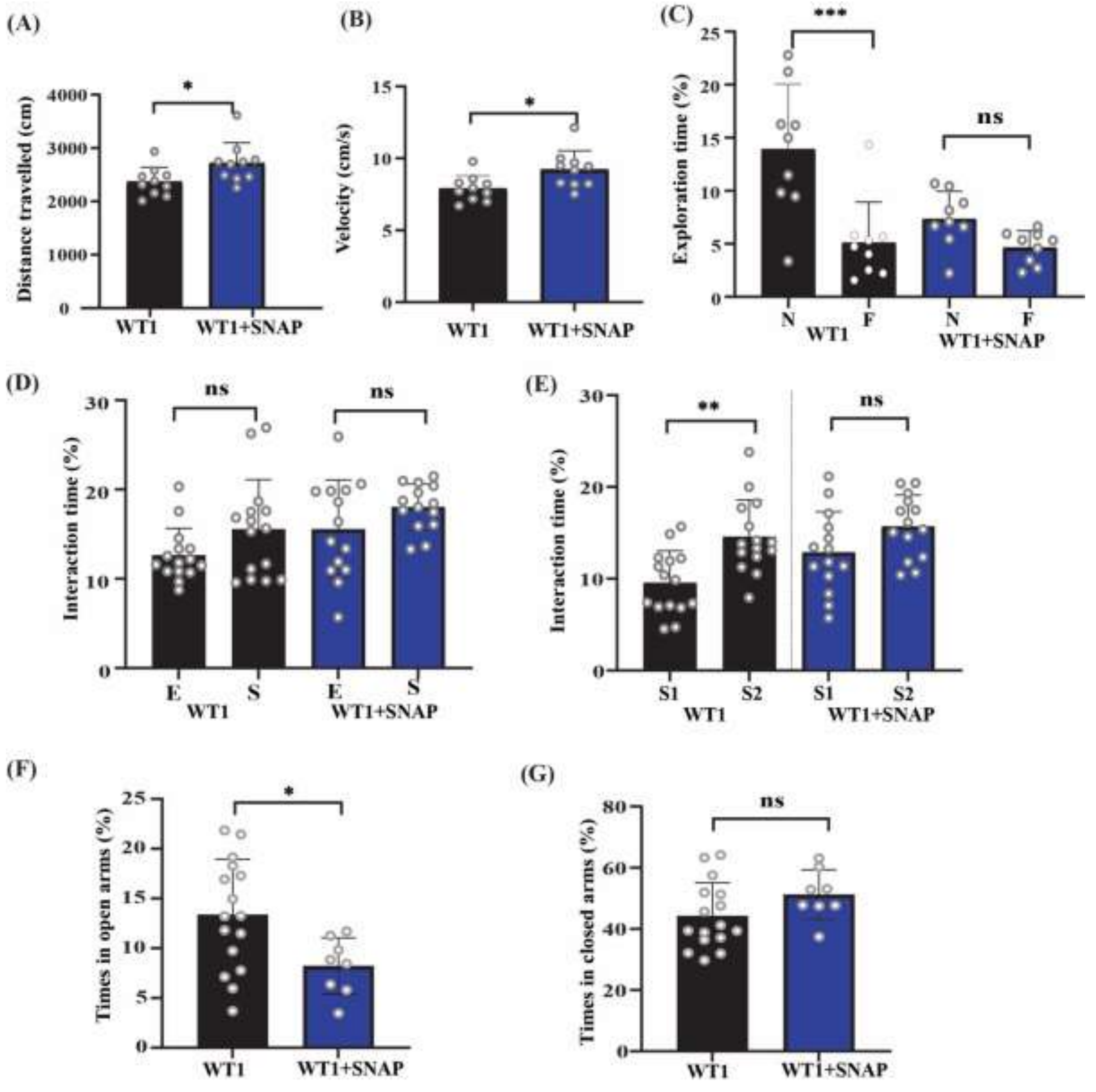

(A)
Distance travelled (cm)
4000
3000
2000
1000
0
*
WT1
WT1+SNAP
(B)
Velocity (cm/s)
15
10
5
0
*
WT1
WT1+SNAP
(C)
Exploration time (%)
25
20
15
10
5
0
***
ns
N
F
WT1
N
F
WT1+SNAP
(D)
Interaction time (%)
30
20
10
0
ns
ns
E
S
WT1
E
S
WT1+SNAP
(E)
Interaction time (%)
30
20
10
0
**
ns
S1
S2
WT1
S1
S2
WT1+SNAP
(F)
Times in open arms (%)
25
20
15
10
5
0
*
WT1
WT1+SNAP
(G)
Times in closed arms (%)
80
60
40
20
0
ns
WT1
WT1+SNAP

Fig S9. SNAP treatment leads to behavioral abnormalities in WT female mice

Panel A & B: Statistical analysis of the motor activity in female WT (n=10), and WT+SNAP (n=10). **Panel C:** Statistical analysis of the exploration time of a novel (N) and a familiar (F) object among the female mice of WT (n=9), and WT+SNAP (n=9). **Panel D:** Statistical analysis of time interacting with either an empty (E) cage or stranger (S) mouse among the female mice in WT (n=15), and WT+SNAP (n=14). **Panel E:** Statistical analysis of time interacting with a familiar (S1) or a novel (S2) mouse among the female mice in WT (n=15), and WT+SNAP (n=14). **Panel F:** Statistical analysis of time spent in open or **(G)** closed arms among the female mice in WT (n=16), and WT+SNAP (n=8). In all tests, the time is presented as the percentage of the total time. In all tests, the data is presented as mean ± SD. Statistical significance was determined using a Two-way ANOVA with Bonferroni's multiple comparisons tests or two-tailed t-test. *$P < 0.05$, **$P < 0.001$, ***$P < 0.0001$, and ns=non-significant**.**

Fig S10.

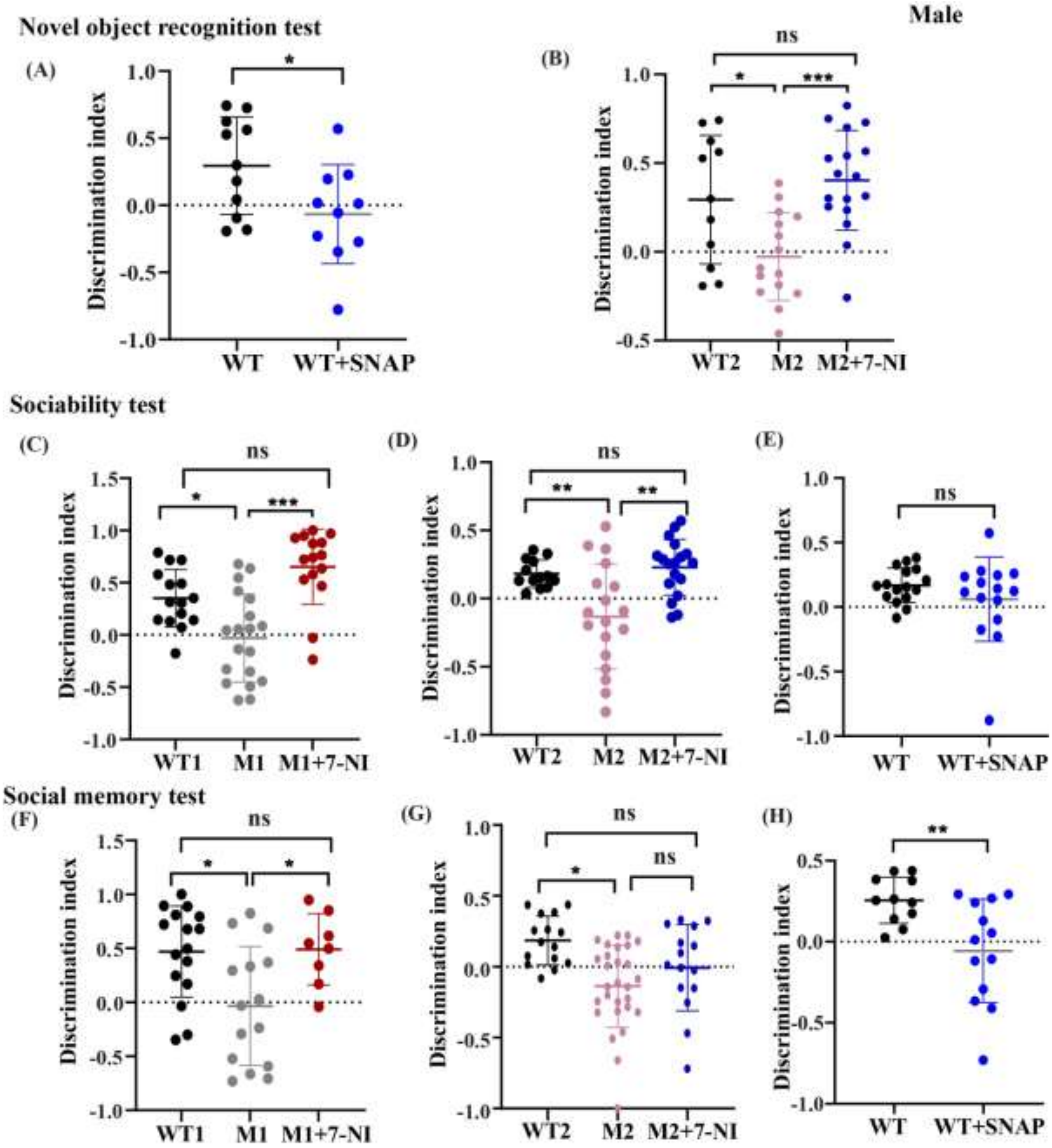
Novel object recognition test
Male
(A)
(B)
Discrimination index
WT
WT+SNAP
WT2
M2
M2+7-NI
ns
Sociability test
(C)
(D)
(E)
WT1
M1
M1+7-NI
Social memory test
(F)
(G)
(H)

Fig S10. Discrimination index of NOR and Sociability tests of male mice

I.For the NOR test the DI was calculated as the difference in exploring the novel and the familiar object divided by the total time exploring both objects:

$$\boldsymbol{DI} = \frac{\boldsymbol{t}\ (\boldsymbol{novel\ object}) - \boldsymbol{t}\ (\boldsymbol{familiar\ object})}{\boldsymbol{t}\ (\boldsymbol{novel\ object}) + \boldsymbol{t}\ (\boldsymbol{familiar\ object})}$$

II. DI in the three-chamber sociability test was calculated as:

$$\boldsymbol{DI}\ (\boldsymbol{day1}) = \frac{\boldsymbol{t}\ (\boldsymbol{stranger\ mouse}) - \boldsymbol{t}\ (\boldsymbol{empty\ cage})}{\boldsymbol{t}\ (\boldsymbol{stranger\ mouse}) + \boldsymbol{t}\ (\boldsymbol{empty\ cage})}$$

$$\boldsymbol{DI}\ (\boldsymbol{day2}) = \frac{\boldsymbol{t}\ (\boldsymbol{novel\ mouse}) - \boldsymbol{t}\ (\boldsymbol{familiar\ mouse})}{\boldsymbol{t}\ (\boldsymbol{novel\ mouse}) + \boldsymbol{t}\ (\boldsymbol{familiar})}$$

DI can have 3 scores; a positive score that indicates more time exploring the novel object or more time interacting with the stranger mouse (day1) or the novel mouse (day2), a negative score that indicates more time exploring the familiar one, empty cage (day1), or the familiar mouse (day2), and a zero score indicates a null preference for either the novel or the familiar or interacting with either the empty or a stranger mouse.

Panel A: DI for exploring the novel object over the familiar object in the NOR test for the WT, and WT+SNAP group. **Panel B:** DI for exploring the novel object over the familiar object in the NOR test for the WT2, M2, and M1+7N2 group. **Panel C:** DI of the preference of the mice to interact with the stranger mouse over the empty cage in the WT1, M1, and M1+7-NI. **Panel D:** DI of the preference of the mice to interact with the stranger mouse over the empty cage in the WT2, M2, and M2+7-NI. **Panel E:** DI of the preference of the mice to interact with the stranger mouse over the empty cage in the WT and WT+SNAP. **Panel F:** DI of the preference of the mice to interact with a novel mouse over a familiar one in the WT1, M1, and M1+7-NI. **Panel G:** DI of the preference of the mice to interact with a novel mouse over a familiar one in the WT2, M2, and M2+7-NI. **Panel H:** DI of the preference of the mice to interact with a novel mouse over a familiar one in the WT and WT+SNAP. Statistical

significance was determined using One-way ANOVA with Bonferroni's multiple comparisons tests or two-tailed t-test. *P < 0.05, **P < 0.001, ***P < 0.0001, and ns= non-significant.

Fig. S11.

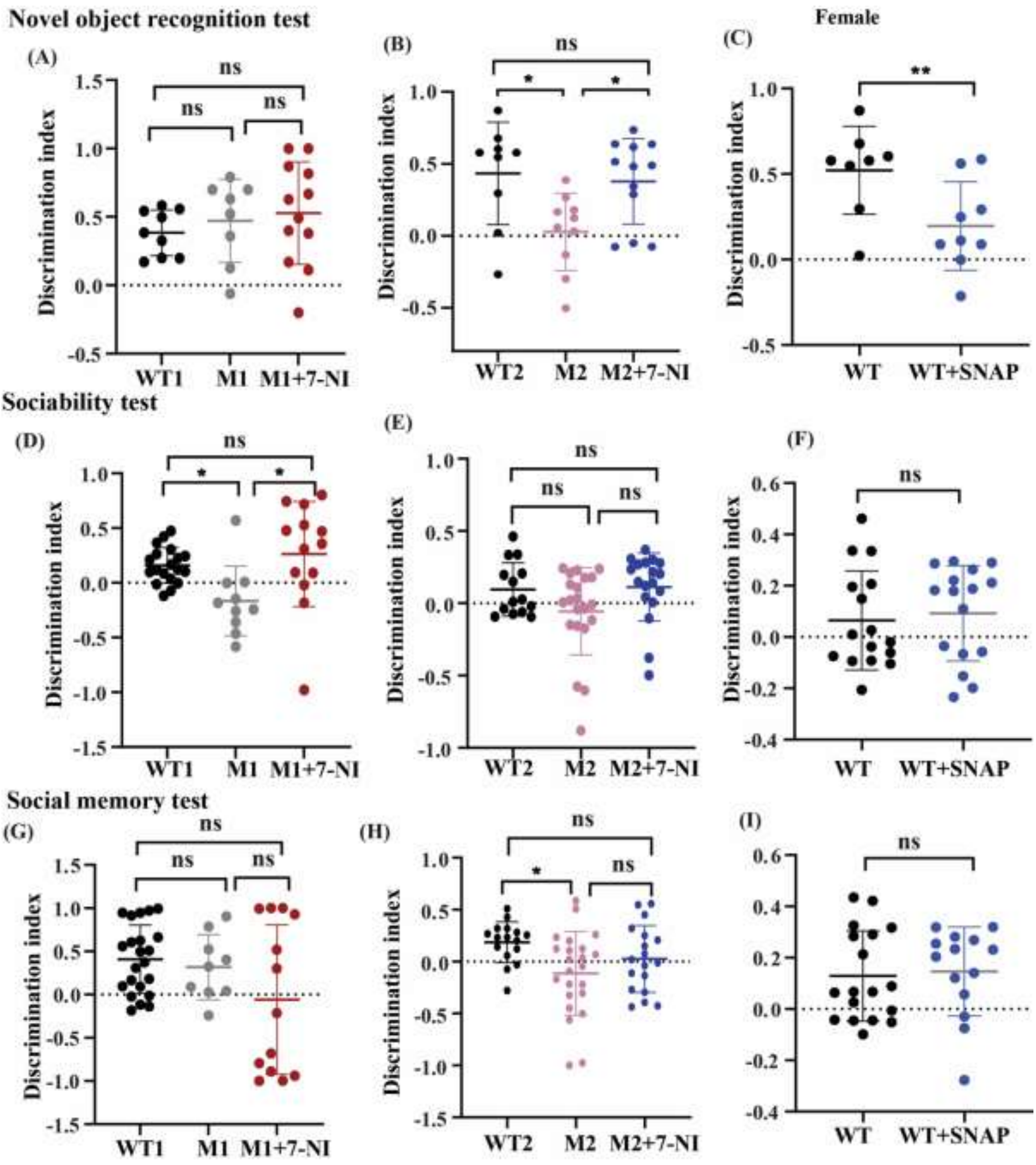
Novel object recognition test
Female
(A)
(B)
(C)
Discrimination index
ns
*
**
WT1
M1
M1+7-NI
WT2
M2
M2+7-NI
WT
WT+SNAP
Sociability test
(D)
(E)
(F)
Social memory test
(G)
(H)
(I)

Fig. S11. Discrimination index of NOR and Sociability tests of female mice

Panel A: DI for exploring the novel object over the familiar object in the NOR test for the WT1, M1, and M1+7-NI groups. **Panel B:** DI for exploring the novel object over the familiar object in the NOR test for the WT2, M2, and M2+7-NI groups. **Panel C:** DI for exploring the novel object over the familiar object in the NOR test for the WT, and WT+SNAP group.

Panel D: DI of the preference of the mice to interact with the stranger mouse over the empty cage in the WT1, M1, and M1+7--NI. **Panel E:** DI of the preference of the mice to interact with the stranger mouse over the empty cage in the WT2, M2, and M2+7-NI. **Panel F:** DI of the preference of the mice to interact with the stranger mouse over the empty cage in the WT and WT+SNAP. **Panel G:** DI of the preference of the mice to interact with a novel mouse over a familiar one in the WT1, M1, and M1+7-NI. **Panel H:** DI of the preference of the mice to interact with a novel mouse over a familiar one in the WT2, M2, and M2+7-NI. **Panel I:** DI of the preference of the mice to interact with a novel mouse over a familiar one in the WT and WT+SNAP. Statistical significance was determined using One-way ANOVA with Bonferroni's multiple comparisons tests or two-tailed t-test. $^*P < 0.05$, $^{**}P < 0.001$, $^{***}P < 0.0001$, and ns= non-significant

Fig. S12.

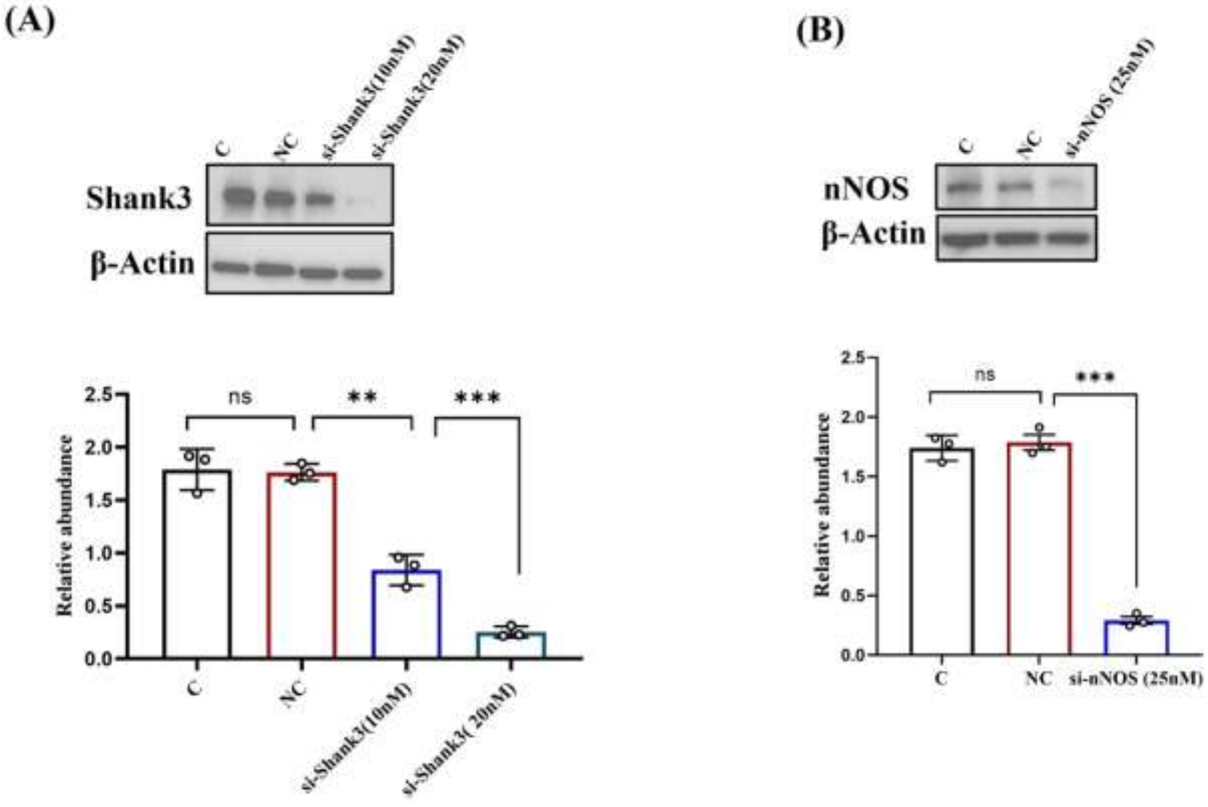

Fig. S12. Transfection of SHSY5Y with si-Shank3 and si-nNOS reduces its protein expression

Panel A: Representative western blot and statistical analysis for Shank3 si-RNA transfected (n=3). **Panel B:** Representative western blot and statistical analysis for nNOS si-RNA transfected (n=3). The data is presented as mean ± SD. Statistical significance was determined using One-way or Two-way ANOVA with Bonferroni's multiple comparisons. ***$P < 0.0001$, and ns=non-significant. Abbreviation- C= control group, NC= negative control, si-Shank3 (10nM) = SHSY5Y cells treated with 10nM of Shank3 siRNA. si-Shank3 (20nM) = SHSY5Y cells treated with 20nM of Shank3 siRNA. si-nNOS= SHSY5Y cells treated with 25nM of nNOS siRNA

Fig. S13.

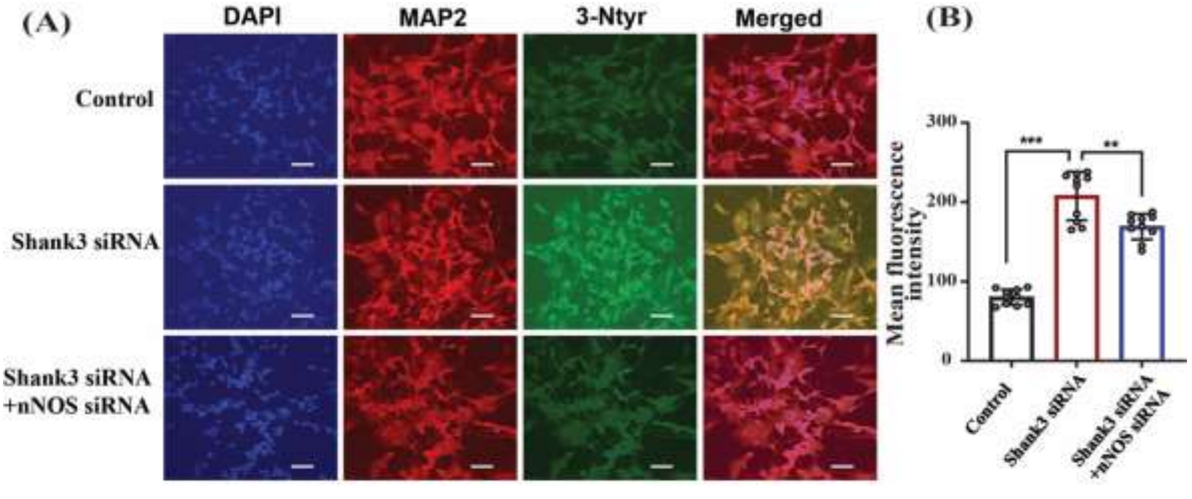

Fig. S13. nNOS knock down in human cell lines reduces nitrosative stress

Representative confocal images of MAP2 (red), 3-Ntyr (red), and DAPI (blue) in SHSY5Y (n=10); SHSY5Y+*siSHANK3* (n=10); and SHSY5Y+*siSHANK3*+*si-nNOS* (n=10). Image captured at 60 x, scale bar represents 50 µm. **Panel B:** Statistical analysis of the mean fluorescence intensity of 3-Ntyr protein. The data is presented as mean ± SD. A one-way ANOVA test with the Tukey post hoc test was used for multiple comparisons in all groups $^*P < 0.05$, $^{**}P < 0.001$, $^{***}P < 0.0001$, and ns=non-significant.
