# Supplementary material for: The NO Answer for Autism Spectrum Disorder
Source: Adv Sci (Weinh). 2023 May 22;10(22):2205783. doi: 10.1002/advs.202205783 (PMC10401098; doi:10.1002/advs.202205783)
Supplement: Supplementary file 2 — Supplemental Table 1 [file ADVS-10-2205783-s002.pdf]

ADVANCED SCIENCE
Open Access

Supporting Information

for *Adv. Sci.*, DOI 10.1002/advs.202205783

The NO Answer for Autism Spectrum Disorder

*Manish Kumar Tripathi, Shashank Kumar Ojha, Maryam Kartawy, Wajeha Hamoudi, Ashwani Choudhary, Shani Stern, Adi Aran and Haitham Amal**

MTT assay for 7-NI in mouse primary neurons

Concentration (uM)	Cell viability (%)
0	100
5	95.1937546
10	91.13763648
20	79.57458845
50	61.70277762
100	50.62171183
250	44.70441817
500	34.40176501
1000	21.99355094
