# Supplementary material for: The NO Answer for Autism Spectrum Disorder
Source: Adv Sci (Weinh). 2023 May 22;10(22):2205783. doi: 10.1002/advs.202205783 (PMC10401098; doi:10.1002/advs.202205783)
Supplement: Supplementary file 3 — Supplemental Table 2 [file ADVS-10-2205783-s004.pdf]

ADVANCED SCIENCE
Open Access

Supporting Information

for *Adv. Sci.*, DOI 10.1002/advs.202205783

The NO Answer for Autism Spectrum Disorder

*Manish Kumar Tripathi, Shashank Kumar Ojha, Maryam Kartawy, Wajeha Hamoudi, Ashwani Choudhary, Shani Stern, Adi Aran and Haitham Amal**

Table 2: Clinical characteristics of the participants (TD and ASD).

TD/ASD	label (plasma)	age (months)	sex (1=boy 2=girl)	cognitive impairment
TD1	C1P	59	1	-
TD2	C2P	38	1	-
TD3	C3P	64	1	-
TD4	C4P	62	1	-
TD5	25-P	68	1	-
TD6	27-P	25	1	-
TD7	28-P	30	2	-
TD8	29-P	24	2	-
TD9	30-P	57	2	-
TD10	43-P (2)	77	1	-
TD11	45-p(2)	82	1	-
TD12	50-P (2)	46	2	-
TD13	55-p	40	1	-
TD14	56-p	49	2	-
TD15	57-p	78	1	-
TD16	58-p	72	1	-
TD17	59-p	28	1	-
TD18	60-p	52	1	-
TD19	61-p	65	1	-
TD20	73-p	80	1	-
ASD1	3P	58	1	mild-moderate
ASD2	SB	52	1	non
ASD3	4P	38	1	moderate
ASD4	1947	66	1	mild-moderate
ASD5	10595	72	2	moderate-severe
ASD6	DN	66	1	mild-moderate
ASD7	8990	66	2	mild-moderate
ASD8	8467	72	2	moderate-severe
ASD9	75-p	40	2	None
ASD10	80-p	76	1	None
ASD11	95-p	25	1	Moderate
ASD12	96-p	52	1	Moderate
ASD13	71-p	27	1	None
ASD14	62-p(3)	34	2	Moderate
ASD15	63-p	47	1	Moderate
ASD16	64-p	48	1	None
ASD17	53-p	45	1	Mild
ASD18	54-p(2)	74	1	Mild
ASD19	42-p(3)	47	1	moderate to severe
ASD20	40-p(2)	44	1	non
