# Supplementary material for: The NO Answer for Autism Spectrum Disorder
Source: Adv Sci (Weinh). 2023 May 22;10(22):2205783. doi: 10.1002/advs.202205783 (PMC10401098; doi:10.1002/advs.202205783)
Supplement: Supplementary file 4 — Supplemental Table 3 [file ADVS-10-2205783-s003.pdf]

ADVANCED SCIENCE
Open Access

Supporting Information

for *Adv. Sci.*, DOI 10.1002/advs.202205783

The NO Answer for Autism Spectrum Disorder

*Manish Kumar Tripathi, Shashank Kumar Ojha, Maryam Kartawy, Wajeha Hamoudi, Ashwani Choudhary, Shani Stern, Adi Aran and Haitham Amal**

Supp. Table 3: 3- Nitrotyrosine relative value in plasma TD and ASD.

**TD/ASD**	**age (months)**	**cognitive impairment**	**3-Ntyr (relative abundance (a.u.))**
TD2	38	-	0.479026
TD3	64	-	0.240735
TD6	25	-	0.567881
TD8	24	-	0.6592
TD11	82	-	0.413949
TD13	40	-	0.724327
TD14	49	-	0.679026
TD20	80	-	0.567881
ASD3	38	moderate	0.852915
ASD4	66	mild-moderate	1.329478
ASD5	72	moderate-severe	1.7552915
ASD9	40	none	0.5601301
ASD12	52	moderate	1.395204
ASD17	45	Mild	0.899478
ASD18	74	Mild	0.901301
ASD19	47	moderate to severe	1.626625
